# Evolution of pathogenicity-associated genes in *Rhizoctonia solani* AG1-IA by genome duplication and transposon-mediated gene function alterations

**DOI:** 10.1186/s12915-023-01526-0

**Published:** 2023-02-01

**Authors:** Aleena Francis, Srayan Ghosh, Kriti Tyagi, V. Prakasam, Mamta Rani, Nagendra Pratap Singh, Amrita Pradhan, R. M. Sundaram, C. Priyanka, G. S. Laha, C. Kannan, M. S. Prasad, Debasis Chattopadhyay, Gopaljee Jha

**Affiliations:** 1 National Institute of Plant Genome Research, Aruna Asaf Ali Marg, New Delhi, 110067 India; 2 Present address: Department of Biosciences, Durham University, Durham, UK; 3 ICAR-Indian Institute of Rice Research (ICAR-IIRR), Rajendranagar, Hyderabad, 500 030 India

**Keywords:** Genome sequence, Fungal pathogenesis, Neofunctionalization, *Rhizoctonia solani*, Sheath blight disease, Transposable elements, Whole-genome duplication

## Abstract

**Background:**

*Rhizoctonia solani* is a polyphagous fungal pathogen that causes diseases in crops. The fungal strains are classified into anastomosis groups (AGs); however, genomic complexity, diversification into the AGs and the evolution of pathogenicity-associated genes remain poorly understood.

**Results:**

We report a recent whole-genome duplication and sequential segmental duplications in AG1-IA strains of *R. solani*. Transposable element (TE) clusters have caused loss of synteny in the duplicated blocks and introduced differential structural alterations in the functional domains of several pathogenicity-associated paralogous gene pairs. We demonstrate that the TE-mediated structural variations in a glycosyl hydrolase domain and a GMC oxidoreductase domain in two paralogous pairs affect the pathogenicity of *R. solani*. Furthermore, to investigate the association of TEs with the natural selection and evolution of pathogenicity, we sequenced the genomes of forty-two rice field isolates of *R. solani* AG1-IA. The genomic regions with high population mutation rates and with the lowest nucleotide diversity are enriched with TEs. Genetic diversity analysis predicted the genes that are most likely under diversifying and purifying selections. We present evidence that a smaller variant of a glucosamine phosphate N-acetyltransferase (GNAT) protein, predicted to be under purifying selection, and an LPMP_AA9 domain-containing protein, predicted to be under diversifying selection, are important for the successful pathogenesis of *R. solani* in rice as well as tomato.

**Conclusions:**

Our study has unravelled whole-genome duplication, TE-mediated neofunctionalization of genes and evolution of pathogenicity traits in *R. solani* AG1-IA. The pathogenicity-associated genes identified during the study can serve as novel targets for disease control.

**Supplementary Information:**

The online version contains supplementary material available at 10.1186/s12915-023-01526-0.

## Background

*Rhizoctonia solani*, a basidiomycetes necrotrophic fungal pathogen, infects a broad range of plant species, including several economically important crops, such as rice, tomato, potato, maize, barley and turf grass [1–3]. The polyphagous nature enables it to survive several years in the soil, even without a primary host. *R. solani* strains have been classified into 14 different anastomosis groups (AGs), i.e. AG1 (which is further divided into intraspecific groups: IA, IB, IC, ID, IF and IE) to AG13 and AGBI [1, 4]. Although placed in the same taxonomic group, strains belonging to different AGs are mostly sexually incompatible. *R. solani* strains exhibit large morphological and pathological diversity [5, 6], and they also differ in karyotype banding pattern, number of nuclei and chromosome number per somatic cells [1, 7]. These features emphasize the need to understand the genomic diversity and evolutionary relationship between *R. solani* strains belonging to different AGs.

The genomes of AG1-IA [8–10], AG1-IB [11], AG2-2IIIB [12], AG3 [13] and AG8 [14] strains of *R. solani* have been sequenced. These studies have catalogued various pathogenicity-associated genes, PHI-base homologues, effectors, cell wall-degrading enzymes (CAZymes) and secondary metabolites encoded in different *R. solani* genomes. Also, the core genes conserved in different AGs and unique genes present in a particular AG strain have been identified [12, 14]. Recent analyses have suggested that the AG1-IA strains are quite diverse from other AGs [9, 15]. They are enriched in homogalacturonan/pectin modification genes and pathogenicity-associated gene families [7, 9].

High degree of heterozygosity (due to coenocytic nature), nucleotide variations (SNPs, indels), large-scale chromosomal rearrangements (insertion, inversion, and deletion, leading to structural and organizational changes in the chromosome) and presence of accessory/mobile chromosomes are observed in different strains of pathogenic fungi [16–18]. They are enriched in repetitive DNAs [17] which serve as a cradle for the evolution of pathogenicity-associated traits [19]. The transposable elements (TEs) as well as other mobile elements play an important role in the evolution of fungal pathogens [20, 21]. They also modulate host specificity as well as aggressiveness of various strains.

In this study, using long-read sequencing technology, we have assembled the genome of a pathogenic strain of *R. solani* AG1-IA (BRS1, an Indian isolate) that causes sheath blight disease in rice [22]. A database is created to facilitate the interactive analyses of the genome. Computational analysis has unravelled the evolutionary relationship and divergence of different AG strains of *R. solani*, in evolutionary time scale. Moreover, the analysis identified relatively large transposon repertoire and segmental gene duplication events leading to the neofunctionalization of genes in AG1-IA strains. Furthermore, through genome sequencing and comparative sequence analysis, we unravel diversity among *R. solani* AG1-IA isolates (*n* = 42) collected from rice fields across different agro-climatic zones of India. The analyses suggest that exchange of genetic material among AG1-IA isolates does occur under field conditions. Moreover, it led to the identification of genes that are most likely undergoing diversifying and purifying selection in *R. solani*. Using some of the genes predicted to be undergoing diversifying/purifying selection, we establish the importance of neofunctionalization of gene in promoting the pathogenesis of *R*. *solani* in rice as well as tomato.

## Results

### Assembly and annotation of *R. solani* AG1-IA (strain BRS1) genome

**Fig. 1:**
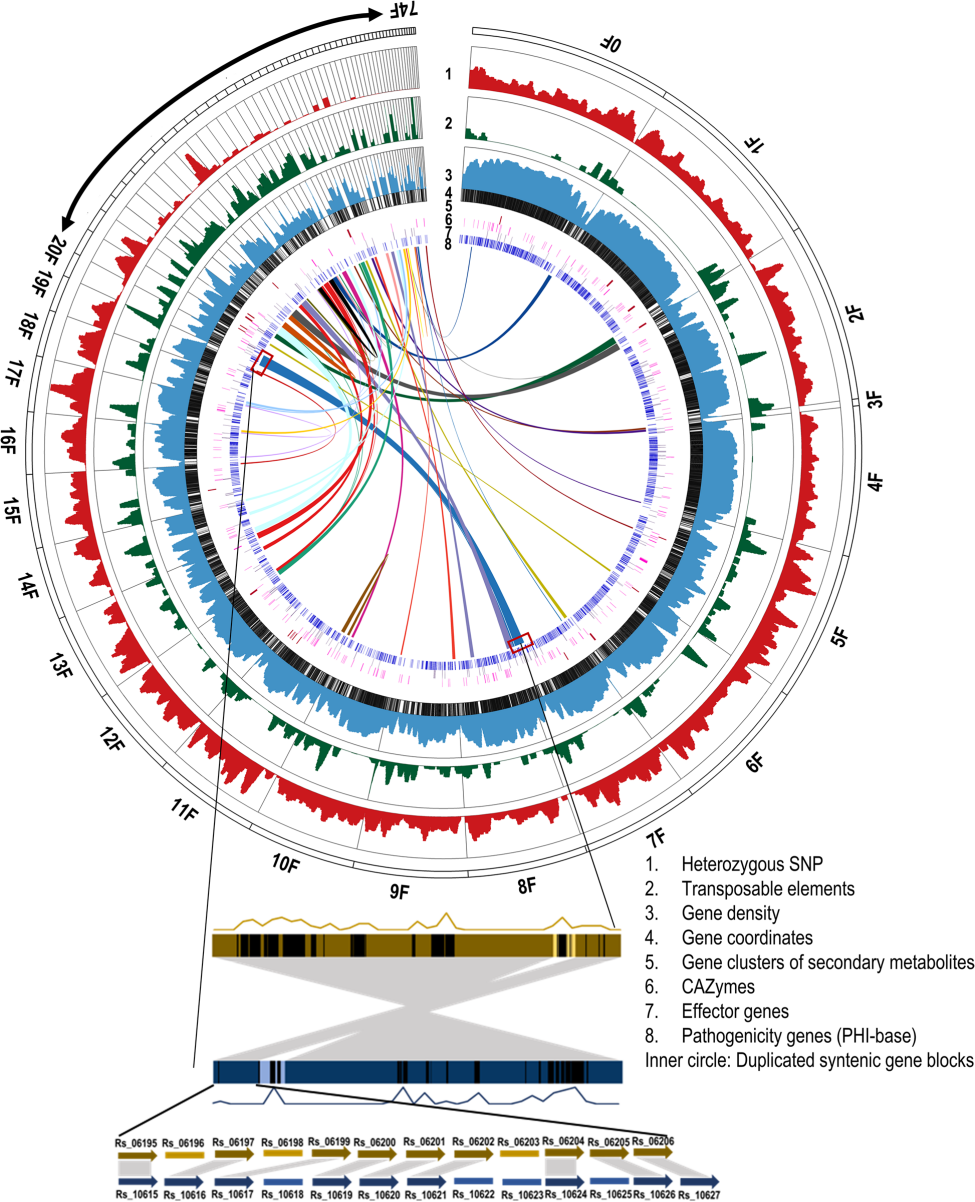
Genomic features of the *R. solani* AG1-IA genome assembly. The Circose diagram showing the density of heterozygous SNPs, genes and transposons in 100 kb with 10-kb sliding windows was presented. The scale used for heterozygous SNPs was 0–3000, for the percentage of transposable elements was 0–100 and for the percentage of protein-coding genes was 0–100. Positions of genes, secondary metabolite-associated gene clusters, CAZymes, effectors genes and PHI-base genes were marked by coloured lines according to their coordinates. Each duplicated syntenic gene blocks are denoted with different colours. Contig names are displayed around the circle. The synteny between two duplicated blocks marked with red boxes is shown below. The density and the coordinates of TEs in each block are shown by the density curve and black lines. Light colours within the duplicated blocks denote non-syntenic regions

The SMRT long-read sequencing platform was used to sequence and assemble the genome of *R. solani* AG1-IA strain BRS1 (pathogenic Indian isolate) [22]. A total of 13.73 Gb sequence data generated by PacBio Sequel II platform was assembled using FALCON and FALCON-Unzip assemblers. These primary contigs were sequence-corrected using about 6 Gb Illumina short-read data following Pilon correction to produce a 44,527,001-bp genome assembly in 74 contigs with an average contig length of 601,716 bp and N50 length of 2,014,351 bp (Additional file 1: Table S1). Sixteen BRS1 contigs were more than 1 Mb in size, and thirty-two contigs covered the assembly of previously reported *R*. *solani* XN genome (41.8 Mb) [10]. Three contigs (total 616 kb) showed sequence homology with the previously sequenced *R*. *solani* B2 genome only. Only 3% of BRS1 assembly did not show sequence homology with the previously reported *R*. *solani* AG1-IA assemblies [8–10]. We observed 0.76% heterozygosity in this assembly, and the heterozygous bases are evenly distributed throughout the genome, irrespective of high- or low-density gene regions (Fig. 1). Annotation of repeat sequences predicted 23.35% interspersed repeat sequences with a vast proportion of retroelements (95% being from Gypsy family) spreading over 10.75% of the assembly (Additional file 2: Table S2). Most of the contigs showed the presence of transposon elements (TEs) being interspersed with the protein-coding genes (Fig. 1). A total of 11,902 high-confidence genes and 14,261 open reading frames (ORFs) (Additional file 3: Table S3) belonging to 7118 unigene and 2448 multigene families with 2 to 10 members per family were annotated. The pathogenicity-associated genes were catalogued using Gene Ontology (GO) search and manual curation (Additional file 4: Table S4). The secondary metabolite encoding gene clusters were identified using the antiSMASH database (Additional file 5: Table S5). Benchmarking Universal Single-Copy Orthologs (BUSCO) analysis [23] showed the presence of 94.1% fungal core genes in the assembly, indicating near completeness of the assembled *R. solani* AG1-IA (BRS1) genome. It is to be noted that BUSCO completeness of previously sequenced AG1-IA strains, XN [10] and B2 [9] genome assemblies, is 89.5% and 81.5%, respectively.

The present *R. solani* AG1-IA genome assembly and protein-coding gene annotation are submitted to the database of National Center for Biotechnology Information (NCBI) with a bioproject ID PRJNA715598 and hosted in a web-based user-interactive rice sheath blight (RSB) database (http:nipgr.ac.in/RSB) having embedded jBrowse genome browser (Additional file 6: Fig. S1). The database provides BLAST options for similarity search for protein-coding genes and comparison of proteins among different AGs of *R. solani*.

### Evolutionary divergence of anastomosis groups in *R. solani*

In order to identify synteny between the BRS1 and XN [10] and B2 [9] genome assemblies, we compared the coordinates of the orthologous protein-coding genes. The analysis reflected a high degree of collinearity between BRS1 and XN/B2 genomes, with thirteen out of total sixteen scaffolds of XN predominantly showing one-to-one alignment with thirteen contigs of BRS1 assembly. A total of 8484 genes (71.28% of total genes) of BRS1 showed collinearity with the XN genes (Additional file 6: Fig. S2A), while 7881 (66.21% of total genes) were colinear with the B2 genes (Additional file 6: Fig. S2B). This suggests that the BRS1 strain is more closely related to XN than B2. We observed significant levels of collinearity between the BRS1 (AG1-IA) and other *R. solani* AGs genome. There were 5865 syntenic genes between AG1-IA and AG8 genome assemblies (Additional file 6: Fig. S3A). AG1-IA (BRS1) genome showed a higher degree of collinearity to AG3 (7513 syntenic genes) (Additional file 6: Fig. S3B) than to AG2-2IIIB (6156 syntenic genes) (Additional file 6: Fig. S3C). On the other hand, potentially due to the fragmented nature of the AG1-IB assembly, least number of syntenic genes (528 syntenic genes) were observed between BRS1 and AG1-IB genome assemblies (Additional file 6: Fig. S3D).

Furthermore, we attempted to delineate unique and shared protein-coding genes among the *R. solani* strains belonging to different AGs. For this, all the predicted proteomes belonging to the respective AGs present in NCBI database were considered. The analysis identified 7300 orthogroups shared between BRS1 and XN/B2 strains of AG1-IA. BRS1 and XN shared 8309 orthogroups, whereas BRS1 and B2 shared 7837 orthogroups, indicating again that BRS1 strain is more closely related to XN than B2 (Additional file 6: Fig. S4A, Additional file 7: Table S6). Notably, 5297 orthogroups comprising 36,554 ORFs were shared between different AGs, while 5896 ORFs belonging to 4654 orthogroups were unique in AG1-IA (Additional file 6: Fig. S4B, Additional file 8: Table S7). We observed 368 unique multi-gene families (*n* = 1610 genes) being present in *R. solani* AG1-IA and previously reported RNA-Seq data [22] supported the expression of 68.49% of the BRS1 specific genes (Additional file 9: Table S8). We further analysed the expression of some of the randomly selected AG1-IA unique genes (*n* = 8) using qRT-PCR and observed majority of them (*Rs_09732*, *Rs_08184*, *Rs_11108*, *Rs_01662* and *Rs_03147*) being upregulated during necrotrophic phase (3 dpi) of infection in rice (Additional file 6: Fig. S5).

**Fig. 2:**
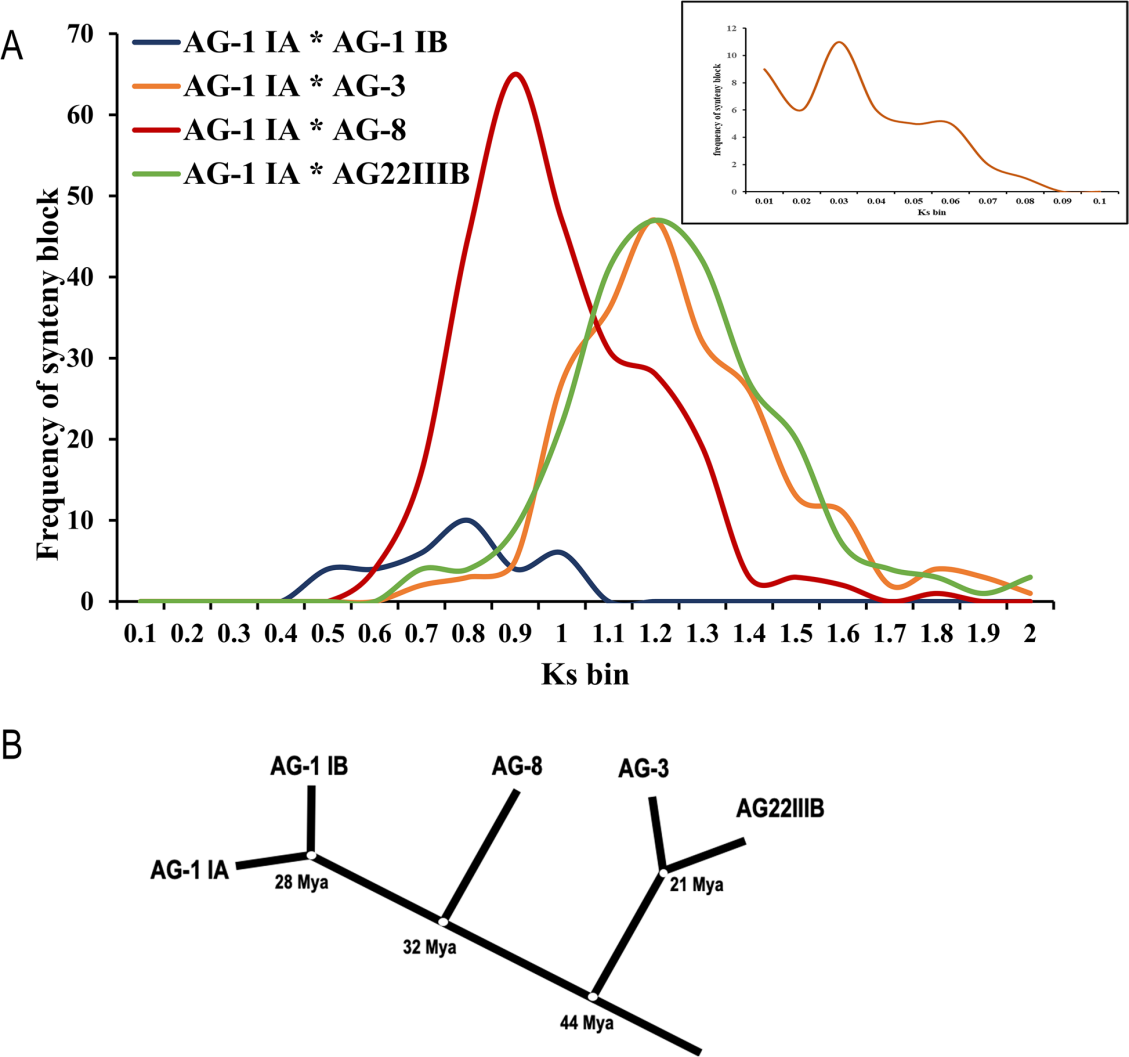
Evolutionary divergence among the AGs of *R. solani*. **A** Distribution of synonymous substitution rates (Ks) of different combinations of orthologous genes of strains of *R. solani*. Ks of the orthologous gene pairs between different strains mentioned were plotted against the number of orthologous gene pairs in different colours. The inset shows the distribution of Ks of the paralogous gene pairs located in the duplicated syntenic gene blocks of the genome assembly of AG1-IA. **B** Phylogenetic tree of five genome-sequenced *R. solani* strains and their divergence times calculated based on Ks of the orthologous gene pairs

To determine the period of divergence of different AGs of *R. solani*, we plotted the rate of synonymous substitution per synonymous site (*K*_*s*_) between the orthogroups in the syntenic blocks between AG1-IA and the other sequenced AGs against the percentage of orthologous pairs (Fig. 2A). The average rate in change of *K*_*s*_ value was considered as 1.3 × 10^−8^ per year, as reported in fungi [24]. The maximum likelihood-based phylogenetic tree constructed using the single-copy orthogroups expectedly predicted AG1-IA and AG1-IB to be phylogenetically closest (Fig. 2B). They seem to have diverged around 28 million years ago (mya). The data further suggested AG1-IA to have diverged from AG8 at about 32 mya. On the other hand, AG3 and AG2-2IIIB had diverged from AG1-IA at around 44 mya, whereas AG3 and AG2-2IIIB had diverged from each other around 21 mya (Fig. 2B).

### Genome duplication in *R. solani*

Genome duplication has been proposed as a strategy for adaptation and evolutionary innovation in fungi. It enhances gene copy number and supplies new genetic material for the emergence of new functions, by mutation and selection [25, 26]. A detailed analysis revealed that about 50% of protein-coding ORFs of AG1-IA (BRS1) have homologues within sequenced *R. solani* strains belonging to different AGs. They are grouped into 2448 (*n* = 7143) multigene families, out of which 38.86% of the ORFs are grouped into two-member gene families. Such a high proportion of duplicated gene pairs prompted an investigation into whether multiple segmental duplications or an ancestral whole-genome duplication (WGD) event had occurred in BRS1 genome. We looked for the existence of any paralogous syntenic blocks as a reminiscence of genome duplication and identified 1338 paralogous genes (10.52% of total genes) in 669 paralogous pairs (duplicates and triplicates). The expression of 70.25% (940 out of 1338 genes) of the paralogous genes were confirmed by the previously reported transcriptome data (Additional file 9: Table S8). They are uniquely grouped into 46 pairs of duplicated syntenic blocks having at least 5 and up to 58 duplicated genes in a block (Additional file 10: Table S9). These duplicated blocks are enriched in genes encoding PHI-base and secreted proteins (*χ*^2^ = 7.83, df = 1, *p =* 0.005). To rule out the possibility that the contigs hosting the syntenic blocks might have arisen due to multinucleated cells, we mapped the long reads as well as the short reads of WGS and the RNA-Seq reads to these contigs. Almost 94% of the long reads, 90% of the short reads and 97.36% of RNA-Seq reads were uniquely mapped on the respective contigs (Additional file 11: Table S10). Apart from that, interspersed genes between the paralogous blocks and the genes flanking these blocks in the corresponding contigs were also different, indicating these contigs to be unique. Together, the duplicated regions covered approximately 15% of the genome assembly and spanned over 55 out of 74 scaffolds. The duplicated genes in each of these regions were in the same order and orientation, providing evidence of an ancestral duplicated state for these regions (Fig. 1). Alternatively, if the 46 duplicated blocks were the resultant of sequential segmental duplication, some of the early duplicated blocks would have been the part of the later events of segmental duplication and according to Poisson distribution would have resulted in 8 triplets within 46 duplicated blocks. We have detected four triplicate blocks with minimum five genes (Additional file 6: Fig. S6) with a moderate probability (*p* = 0.057) indicating sequential segmental duplication along with whole genome duplication might have happened in *R. solani* BRS1.

We analysed the two other genome assemblies of AG1-IA strains, i.e. XN and B2 for duplicated syntenic blocks and identified 484 genes (4.21% of total genes) in B2 genome and 670 genes (5.39% of total genes) in XN genome, arranged in 31 and 20 duplicated syntenic blocks, respectively (Additional file 6: Fig. S7 A, B). Analysis of synonymous substitution rates of the paralogous gene groups predicted the range of duplication periods of BRS1 as 0.76–1.1 mya, XN as 1.32–1.79 mya and B2 as 2.69–3.82 mya. The genome duplication has occurred in all these three AG1-IA strains after the divergence of AG1-IA from AG1-IB (Fig. 2, Additional file 6: Fig S7C). This hypothesis was further corroborated by the observation that, out of 669 paralogous gene pairs in BRS1 (AG1-IA), 620 gene pairs have orthologs in the AG1-IB genome assembly and 88% (547 genes) of those are present as single copy genes (only one ortholog) in AG1-IB genome (Additional file 12: Table S11). It is expected that the duplicated genes are lost over time because of recombinations [27]. Decrease in the number of paralogous genes with the increase in the duplication period in the AG1-IA strains support this hypothesis.

### Transposable elements are associated with genome evolution of *R. solani* AG1-IA

**Fig. 3:**
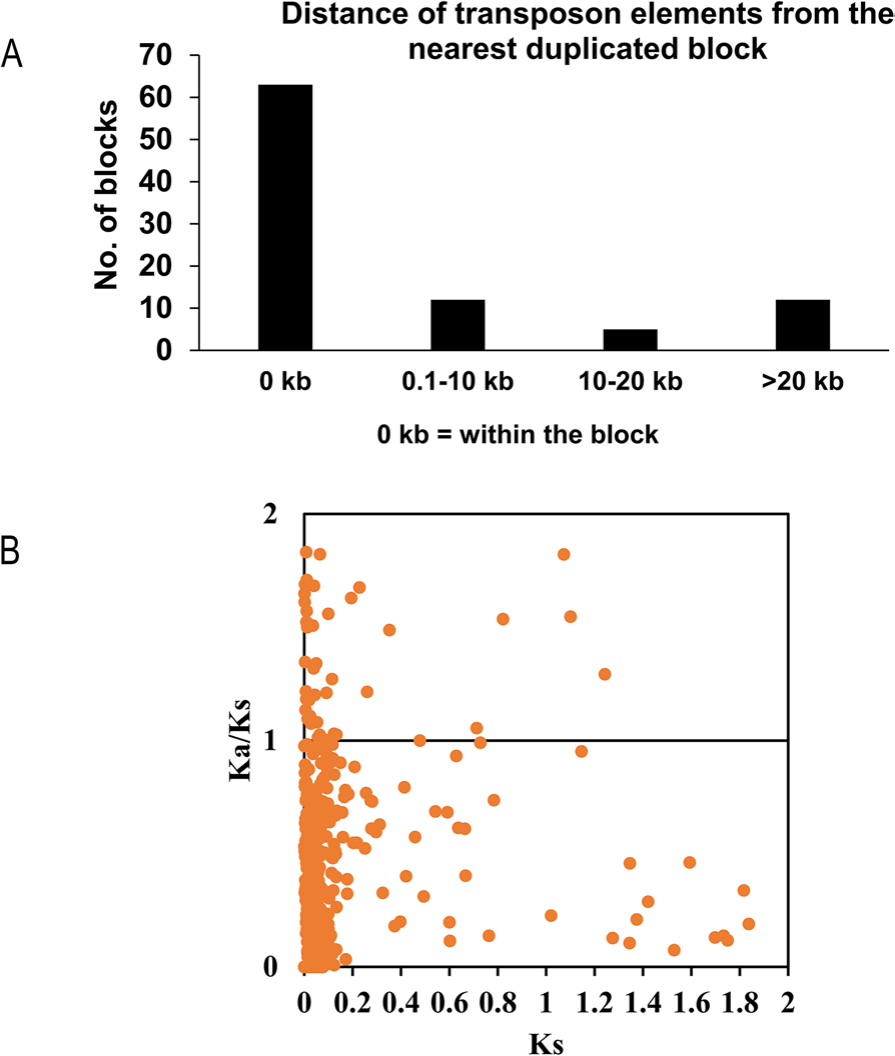
Distribution of TEs in *R. solani* genome and neofunctionalization of *R. solani* genes. **A** Distance of the transposon elements nearest to a duplicated paralogous gene block. A number of paralogous gene blocks were plotted against the distance of the nearest transposon elements. 0 kb denotes where the transposon elements reside within the paralogous blocks. **B** A scatter plot of the ratio of the rates of non-synonymous to synonymous substitutions against the synonymous substitution of the paralogous gene pairs of *R. solani* AG1-IA

Transposable elements (TEs) are abundant in filamentous fungi [28]. They create localized blocks in the duplicated genomic regions by inserting breakpoints and increasing the rate of chromosomal rearrangements, thereby enhancing genomic variations [18, 28]. We noticed the presence of TEs within sixty-two out of total 92 paralogous blocks in BRS1 (Fig. 3A). The density of TE in the duplicated blocks and within the 10-kb regions flanking the duplicated blocks were 1461 bp/10 kb and 1665 bp/10 kb, respectively, which is significantly higher (Wilcoxon signed-rank test, *p* < 0.001) than the overall average of 1120 bp of TE per 10 kb of genome. The appearance of a high frequency of TEs in the duplicated block has introduced disruption in the continuity of synteny. Merging the duplicated blocks that are within 200 kb after discounting the TEs would have extended the duplicated blocks from 15 to 22.1% of the genome assembly. Together, these analyses suggest that whole-genome duplication and TE-mediated gene disruption have profound effect on genome evolution of *R. solani* AG1-IA.

### Neofunctionalization of genes due to gene duplication and transposon elements

To determine whether the gene duplication has led to the emergence of new functions, we calculated the ratio of non-synonymous to synonymous substitution rate (*K*_*a*_/*K*_*s*_) of the TE-associated paralogs and plotted against the synonymous substitution rate (*K*_*s*_). Sixty-four gene pairs were identified to have *K*_*a*_/*K*_*s*_ > 1, suggesting neofunctionalization after gene duplication (Fig. 3B, Additional file 13: Table S12). We identified six paralogous pairs wherein one paralog encodes a potentially secreted protein whereas, the other encodes a non-secreted protein (Additional file 14: Table S13). Among one of such paralog pairs (Rs_09385-Rs_11744), Rs_09385 having a secretion signal showed significantly higher expression level during pathogenesis in rice, compared to Rs_11744 that lacks the secretion signal (Additional file 6: Fig. S8). This suggests *R. solani* AG1-IA has adapted one of the duplicated paralogs as a secreted effector protein to facilitate host colonization. However, in two other cases (Rs_07297-Rs_11800 and Rs_11307-Rs_11399), the paralog with secretion signal exhibited relatively less expression during pathogenesis in rice, compared to the paralog without secretion signal (Additional file 6: Fig. S8). The downregulated expression of such secreted paralogs might facilitate the pathogen to avoid host recognition and induction of defence responses.

**Fig. 4:**
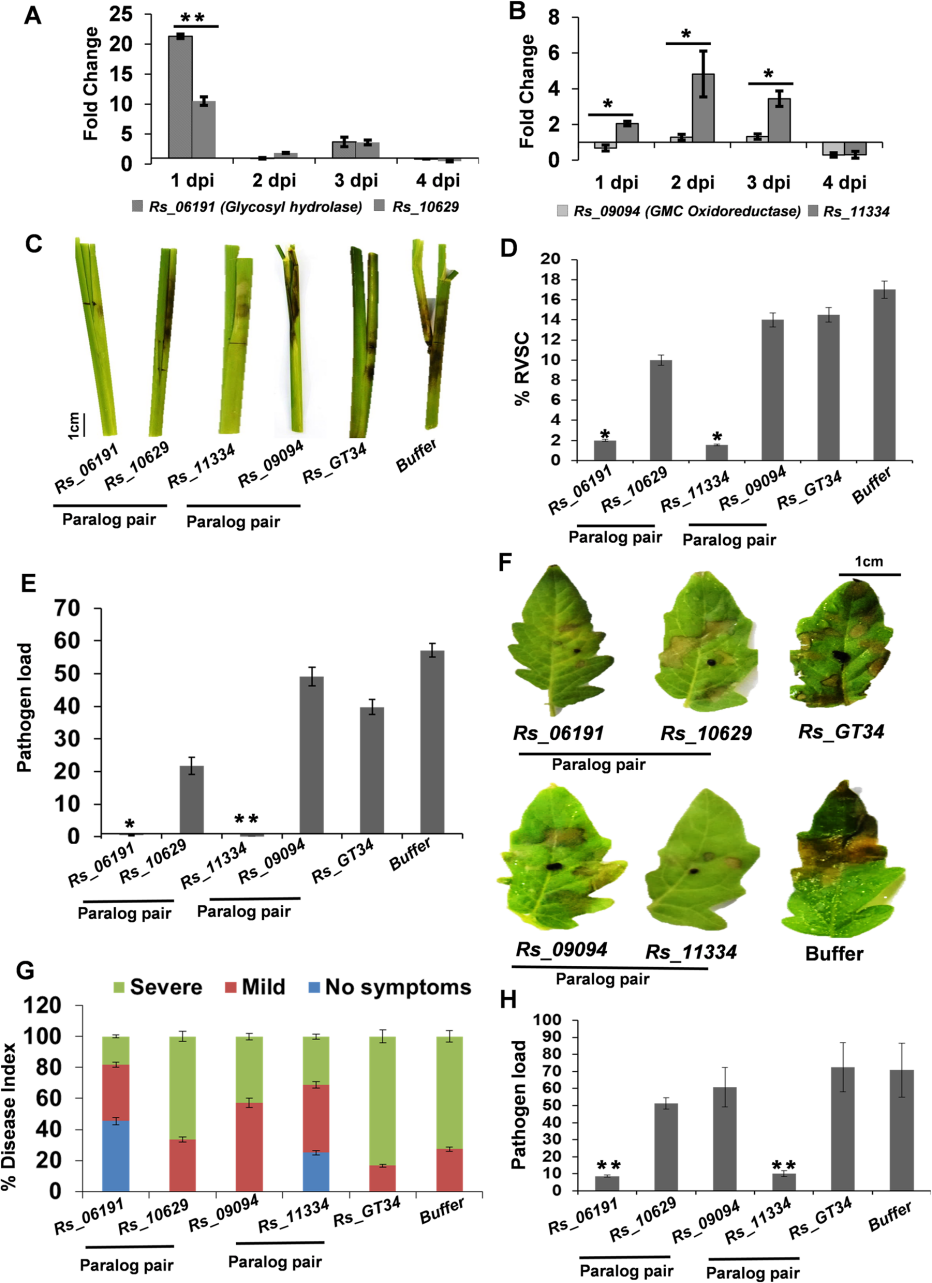
Loss and gain of domain in paralog gene pairs modulate the pathogenesis of *R. solani* AG1-IA. qRT-PCR-based expression analysis (2^−ΔΔCt^) of **A** paralog pair (*Rs_06191*; with glycosyl hydrolase domain and *Rs_10629*; lacking the domain) and **B** Paralog pair (*Rs_09094*; with GMC oxidoreductase domain and *Rs_11334*; lacking the domain) upon *R. solani* infection in rice, at different time points. The fold change in the gene expression was estimated with respect to 0 dpi, using *18S rRNA* of *R. solani* for normalization. **C** Disease symptoms, **D** disease index (% RVSC) and **E** pathogen load in rice tillers infected with gene-silenced (dsRNA-treated) and buffer-treated (control) *R. solani* mycelia, at 3 dpi. **F** Disease symptoms, **G** disease index and **H** pathogen load in tomato leaves infected with gene silenced (dsRNA-treated) and buffer-treated (control) *R. solani* mycelia, at 3 dpi. The *Rs_GT34*-silenced and buffer-treated *R. solani* were used as a negative control. The pathogen load (2^−ΔCt^) was estimated as an abundance of 18S rRNA of *R. solani*, upon normalization with rice *18S* or tomato *actin* gene. The graph shows the mean values ± standard error of three biological replicates. “**”indicates significant difference at *p* ≤ 0.01, and “*” indicates significant difference at *p* ≤ 0.05. GMC oxidoreductase, glucose–methanol–choline oxidoreductases. Scale bar: 1 cm

Out of 669 duplicated gene pairs, we observed 50 pairs to possess at least one different domain altogether and 107 pairs to have a disrupted/missing domain in one of the partners (Additional file 15: Table S14). Notably, 31 of the duplicated paralogous gene pairs exhibited a loss of functional domain in one of the paralogs. For one of such pairs (*Rs_06191*-*Rs_10629*), the paralog with additional domain (*Rs_06191*; glycosyl hydrolase) had significantly higher expression during establishment phase (1 dpi) of pathogenesis in rice, compared to the domain deleted paralog (*Rs_10629*) (Fig. 4A). However, in case of *Rs_09094*-*Rs_11334* duplicated pair, the paralog with domain deletion (*Rs_11334*) exhibited significantly higher expression during necrotrophic (2–3 dpi) phase of *R. solani* pathogenesis in rice, compared to the one (*Rs_09094*; GMC oxidoreductase) with additional domain (Fig. 4B). The differences in the expression of the duplicated gene pairs of *R. solani* during colonization in rice further suggested their neofunctionalization. It is also possible that the duplicated pairs may have host-specific functions and contribute host-specificity in *R. solani*.

Furthermore, to analyse the importance of the genes undergoing neofunctionalization in *R. solani*, we used a dsRNA-based approach [29] to silence *R. solani* genes and study their importance during pathogenesis in rice and tomato. We used gene-specific dsRNA to downregulate two duplicated paralogous gene pairs (*Rs_06191*-*Rs_10629* and *Rs_09094*-*Rs_11334*) of *R. solani*. The qRT-PCR analysis reflected efficient silencing of the target genes during infection in rice (Additional file 6: Fig. S9A) and tomato (Additional file 6: Fig. S9B). Notably, silencing of *Rs_06191* but not *Rs_10629*, whereas silencing of *Rs_11334* but not *Rs_09094*, compromised the pathogenesis of *R. solani* in rice (Fig. 4C–E) as well as tomato (Fig. 4F–H). The disease symptoms (Fig. 4C, F), disease severity index (Fig. 4D, G) and pathogen load (Fig. 4E, H) were significantly reduced in plants infected with *Rs_06191* and *Rs_11334* silenced *R. solani* mycelia, compared to the control plants, infected with buffer-treated mycelia. On the other hand, silencing of *Rs_10629*, *Rs_09094* and a previously reported negative control gene *Rs_GT34* (glycosyl transferase family protein 34; *Rs_04707* in the BRS1 assembly) [29] did not compromise the pathogenesis of *R. solani* (Fig. 4C–H).

Overall, these analyses unravel the importance of TE-mediated gene duplication events, leading to neofunctionalization of genes and evolution of pathogenicity-associated genes in *R. solani* AG1-IA.

### Dynamics of genome evolution and selection of pathogenicity-associated genes in the extant rice field isolates of *R. solani* AG1-IA

We have so far studied and discussed how the historical events of whole and segmental genome duplication and TE-mediated structural reorganization have shaped the genome and pathogenicity of *R. solani* AG1-IA. To further investigate the association of TE with the natural selection and pathogenicity of *R. solani*, we wanted to identify the genomic regions potentially undergoing purifying and diversifying (balancing) selections. For this, we collected forty-two diverse *R. solani* AG1-IA isolates (Additional file 6: Fig. S10; Additional file 16: Table S15) from the rice fields of twelve agro-climatic zones of India [30]. Their genomes were sequenced with 350× coverage (16 Gb data/sample) and identified a total of 5,046,121 single nucleotide polymorphisms (SNPs) by physically mapping the reads on the assembled AG1-IA (BRS1) genome. Phylogenetic and principal component analyses classified the field isolates into three distinct genomic groups and a subgroup of admixture between group I and group II (Additional file 6: Fig. S11A, B; Additional file 17: Table S16). We did not find any correlation between their geographic locations, pathogenicity and genotypes.

**Fig. 5:**
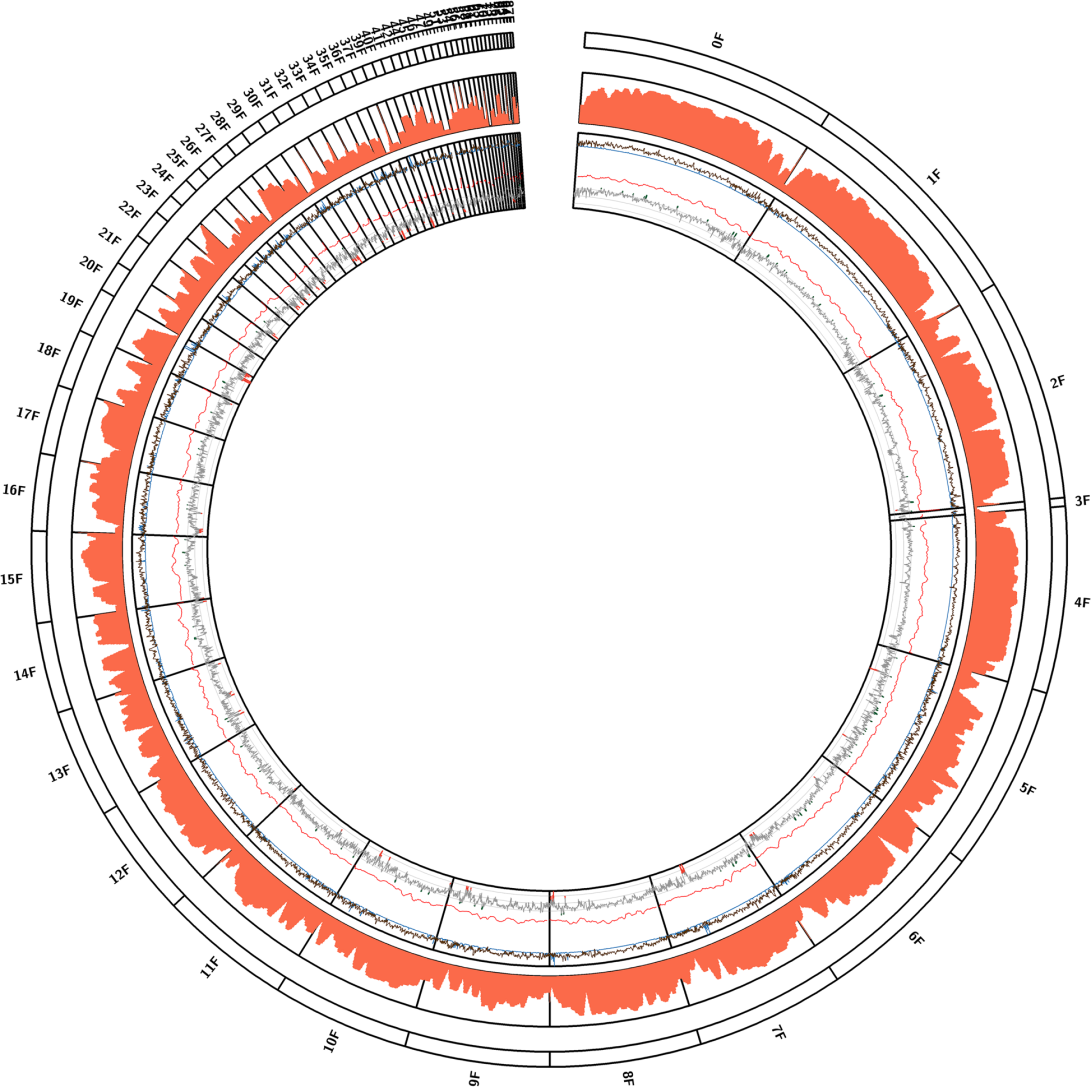
Genomic variations in the Indian isolates of *R. solani* AG1-IA and identification of genomic regions of high and low variations. Diversity metrics, presented as average pair-wise nucleotide diversity *θ*_*π*_ (brown line, min = 0, max = 0.5), *θ*_*⍵*_ (blue line, min = 0, max = 0.5), *F*_ST_ (red line, min = 0, max = 1.0) and Tajima’s *D* (grey line, min = − 3, max = + 3). The regions in the first percentile of Tajima’s *D* distribution denoting purifying selection are highlighted in red, and the 99th percentile regions undergoing balancing selection are highlighted in green colour. The gene density plotted in orange colour. Gene density and *F*_ST_ are plotted in a 100-kb sliding window. The other values are plotted in the 500-SNP sliding window with step size 100 SNP using Circos

**Fig. 6:**
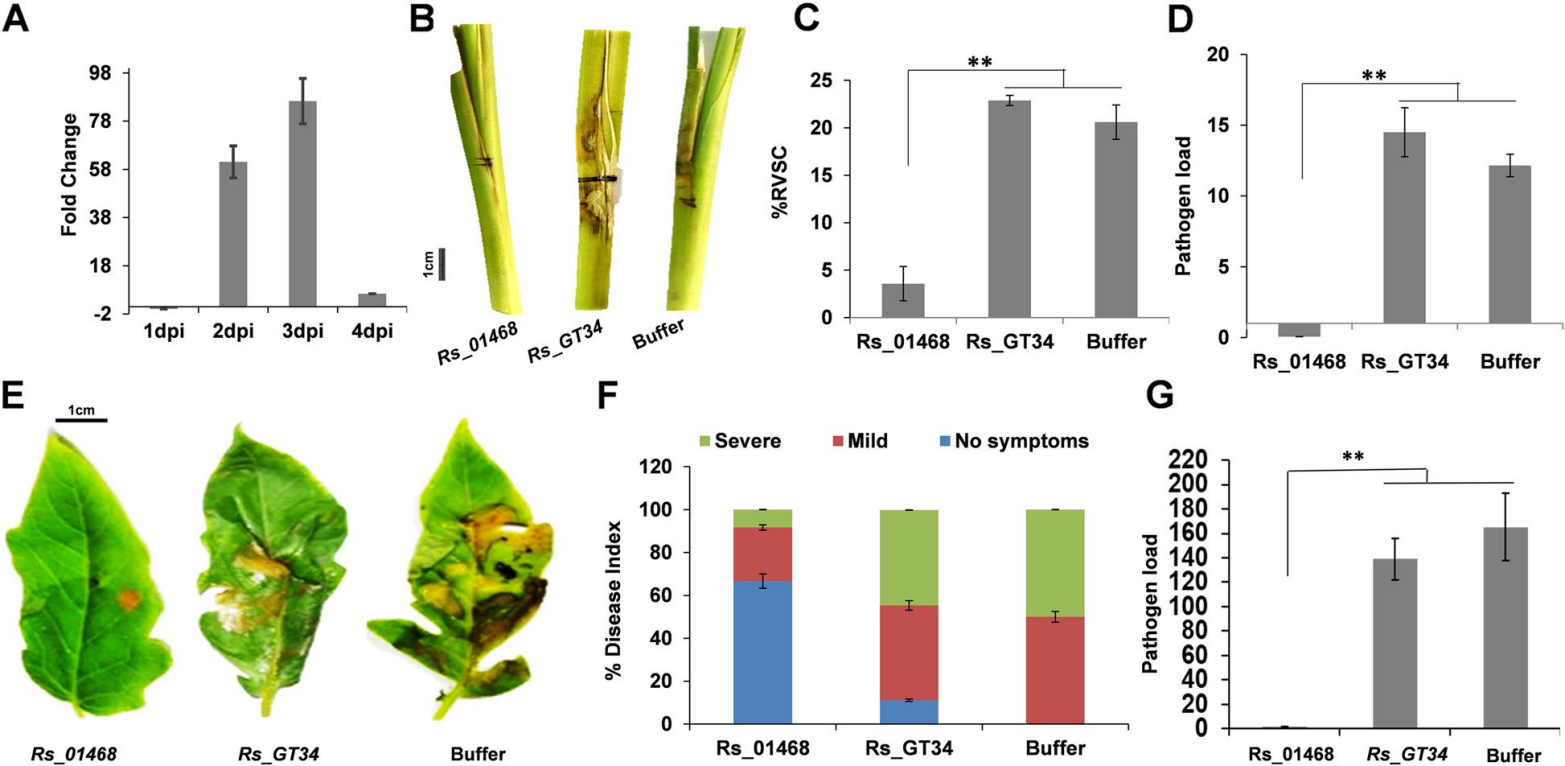
Silencing of *Rs_01468* (LPMO) gene under diversifying selection compromises the pathogenesis of *R. solani*. **A** qRT-PCR-based expression analysis of *Rs_01468* during the pathogenesis of *R. solani* in rice at different time points, compared to 0 dpi. The expression was normalized using *R. solani 18S* rRNA. **B** Disease symptoms, **C** disease index (% RVSC) and **D** pathogen load in rice tillers infected with *Rs_01468* silenced *R. solani* compared to control (*Rs_GT34* silenced and buffer treated), at 3 dpi. **E** Disease symptoms, **F** disease index and **G** pathogen load in tomato leaves infected with *Rs_01468*-silenced *R. solani* compared to control (*Rs_GT34* silenced and buffer treated), at 3 dpi. The pathogen load (2^−ΔCt^) was estimated as an abundance of 18S rRNA of *R. solani*, upon normalization with rice *18S* or tomato *actin* gene. The graph shows the mean values ± standard error of three biological replicates. “*” indicates a significant difference at *p* ≤ 0.01. LPMO, lytic polysaccharide monooxygenase; GT34, glycosyl transferase family 34 protein. Scale bar: 1 cm

To understand the landscape of genomic diversity in the Indian rice field isolates of *R*. *solani*, we calculated the average pairwise nucleotide diversity (*θπ*), Waterson’s estimator of segregating sites (*θ⍵*) and Tajima’s *D* within the total population [31]. Plotting of diversity metrics in sliding windows across the genome revealed a high *θπ* all over the genome. Notably, the high *θ⍵* regions are significantly rich in TE (Wilcoxon signed-rank test, *p* = 0.004) (Fig. 5). We removed the loci with 5% of the minor allele frequency due to probable population contraction [32]. A total of 87 genomic regions containing 437 genes with top 1% Tajima’s *D* values were predicted to be the candidates for the diversifying selection (Additional file 18: Table S17). We analysed the expression of five genes of PHI-base and secreted proteins categories within this top 1% Tajima’s *D* values, upon infection with *R. solani* (Fig. 6A, Additional file 6: Fig. S12). The qRT-PCR analysis showed that a gene (*Rs_01468*) encoding a LPMO_AA9 protein (Lytic polysaccharide monooxygenase_Auxillary activity family 9 domain containing proteins) residing in a genomic region with a high Tajima’s *D* value of 2.59 was highly upregulated during 2–3 dpi of *R. solani* infection in rice (Fig. 6A), which coincides with the transition from establishment (biotrophy) to necrotrophy phase [33]. Interestingly, dsRNA-mediated silencing of the gene compromised the pathogenesis of *R. solani* in rice (Fig. 6B–D) and tomato (Fig. 6E–G). The disease symptoms (Fig. 6B, E), disease severity index (Fig. 6C, F) and pathogen load (Fig. 6D, G) were significantly reduced in plants infected with *Rs_01468* silenced *R. solani* mycelia, compared to those infected with buffer treated (control) or *Rs_GT34* silenced mycelia. Moreover, qRT-PCR analysis reflected that dsRNA treatment was efficient in silencing of the target genes of *R. solani* during pathogenesis (3 dpi) in rice (Additional file 6: Fig. S9C) as well as tomato (Additional file 6: Fig. S9D).

**Fig. 7:**
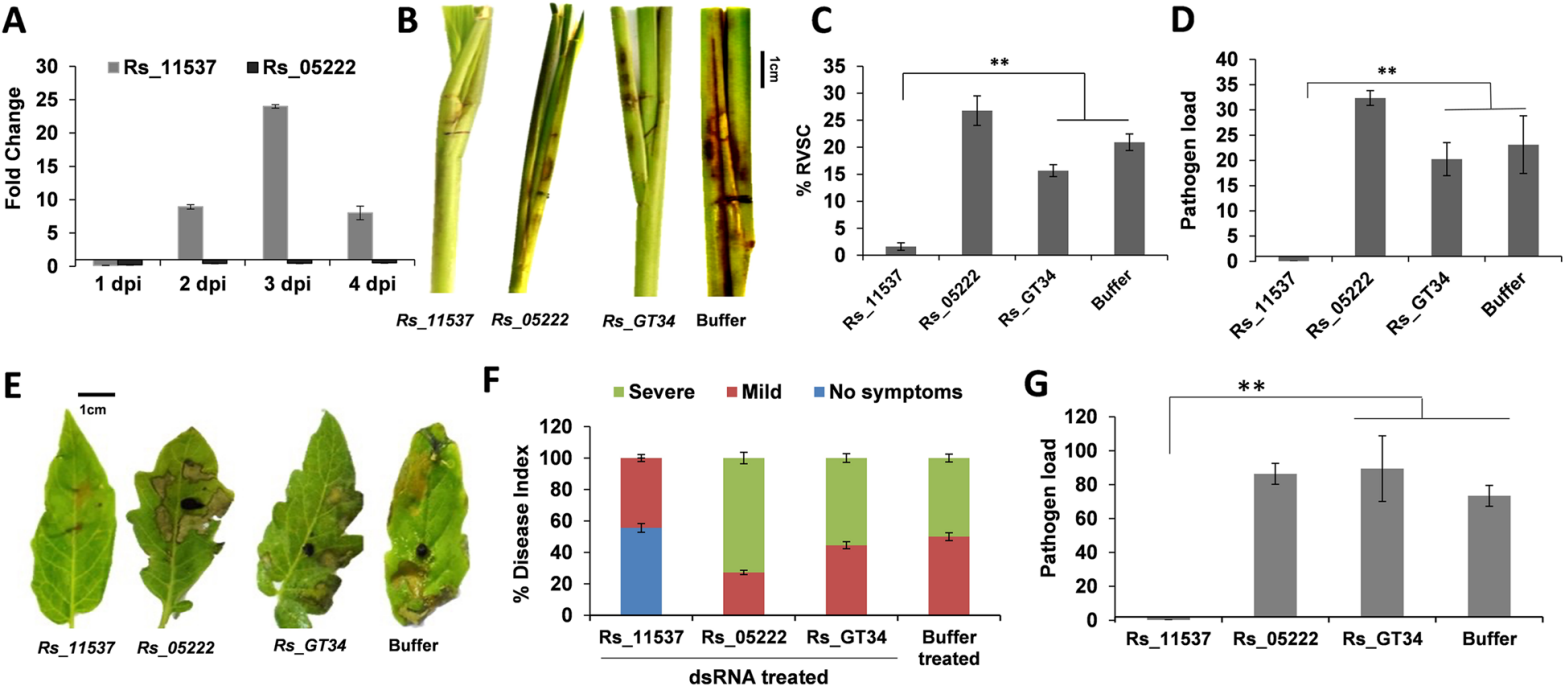
*Rs_11537* (GNAT), a gene under purifying selection is important for the pathogenesis of *R. solani*. **A** qRT-PCR-based expression analysis of *Rs_11537* and *Rs_05222* genes during *R. solani* pathogenesis in rice at different time points, as compared to 0 dpi using rice actin gene for normalization. **B** Disease symptoms, **C** disease index (%RVSC) and **D** pathogen load in rice tillers infected with gene silenced (dsRNA treated) or buffer treated *R. solani*, at 3 dpi. **E** Disease symptoms, **F** disease index and **G** pathogen load in tomato leaves infected with gene-silenced (dsRNA-treated) or buffer-treated *R. solani*, at 3 dpi. The pathogen load (2^−ΔCt^) was estimated as an abundance of 18S rRNA of *R. solani*, upon normalization with rice *18S* or tomato *actin* gene. The graph shows the mean values ± standard error of three biological replicates. Scale bar: 1 cm

On the other hand, 43 genomic regions representing 79 genes within the lowest 1% Tajima’s *D* values were predicted to be the probable targets of purifying selections, and those were located in the significantly TE-enriched regions (Wilcoxon signed-rank test, *p* < 0.001) (Fig. 5, Additional file 18: Table S17). Among the genes likely to be undergoing purifying selection, the expression of a gene *Rs_11537* encoding glucosamine phosphate N-acetyltransferase (GNAT), residing in a genic region with the lowest Tajima’s *D* value of − 5.34, was highly upregulated during 2–3 dpi of *R. solani* infection in rice (Fig. 7A). *R. solani* strains encode large number of GNAT proteins with size ranging from ~ 79–700 aa. Rs_11537 is only 79 aa long and phylogenetic analysis reflected it to be in a clade with a few other smaller size GNAT proteins (two of them were 86 aa long while Rs_05222 and some others were ~183 aa) (Additional file 6: Fig. S13). This tempted us to speculate that the Indian strain of *R. solani* (BRS1) has particularly selected the smaller sized GNAT protein to effectively colonize plants. The qRT-PCR-based gene analysis reflected that the expression of smaller sized (*Rs_11537*) but not a relatively bigger sized (*Rs_05222*) GNAT (which was not under purifying selection) was induced during pathogenesis of *R. solani* in rice (Fig. 7A). Further using the dsRNA approach, we downregulated the *Rs_11537* and *Rs_05222* genes and studied the impact on pathogenesis of *R. solani* in rice (Fig. 7B–D) and tomato (Fig. 7E–G). The qRT-PCR analysis reflected the efficient silencing of the target genes in rice (Additional file 6: Fig. S14A) and tomato (Additional file 6: Fig. S14B). Moreover, no cross-silencing of *Rs_05222* gene was observed upon dsRNA mediated silencing of *Rs_11537* (Additional file 6: Fig. S14C, D). Interestingly, the rice and the tomato plants infected with *Rs_11537* silenced *R. solani* mycelia had significantly reduced necrotic disease lesions (Fig. 7B, E), disease index (Fig. 7C, F) and pathogen load (Fig. 7D, G), compared to those infected with *Rs_05222*/*Rs_GT34* silenced or buffer-treated mycelia. Overall, our data exemplify that genes predicted by genetic diversity analysis to be under purifying selection pressure are located in the TE-rich regions of the genome and contribute towards pathogenesis of *R. solani*.

## Discussion

*Rhizoctonia solani* is an important fungal model system to study the genetic adaptation of the pathogens to colonize a wide range of plant species. We have assembled the genome of an AG1-IA strain (BRS1) utilizing hybrid genome assembly of Pacbio and Illumina reads, and unravelled insights about the diversification of different AGs. The synonymous nucleotide substitution rates within the *R. solani* AGs suggest that diversification of *R. solani* strains into various AGs has happened over the last ~ 44 million years. Similarly, most of the extant crop plants have undergone whole genome duplication (WGD) and diversification over the last 60–66 million years (during the tertiary period of Cenozoic era) to adapt to different ecological niches [34]. Our data suggest that *R. solani* strains diversified into different AGs around similar period to colonize different plant species (members of Solanaceae, Asteraceae, Poaceae) and specialized to infect different plant parts (such as roots, stems and leaves). Notably, the AG3 and AG2-2IIIB strains, which diverged from the other AGs ~ 44 mya predominantly infect underground plant parts, whereas AG1-IA, AG1-IB and AG8, which are closely related and diverged over the last ~ 32 million years, infect aerial plant parts, such as stem and leaves [12, 35, 36].

Evidence of WGD followed by gene family expansion by gene gain and loss has been reported in some of the fungi, including fungal pathogens [18, 26, 37–39]. Ancient WGD event followed by extensive gene deletions played an important role in the evolution of *Saccharomyces cerevisiae*, the baker’s yeast, a member of the division Saccharomycotina [26]. On the other hand, recent WGD events leading to the expansion of gene families have been reported in the members of Mucoromycotina, including *Phycomyces blakesleeanus*, the dung fungus; *Mucor circinelloides*, the opportunistic human pathogen [39]; and *Rhizopus oryzae*, the human pathogen [18]. In these cases, the WGD has contributed towards the expansion of pathogenicity-associated genes and signal transduction pathways for better sensing of environmental stimuli. The gene expansion events have enabled *Rhizopus oryzae* (causal organism of mucormycosis) to utilize complex carbohydrates and rapidly generate energy to ensure a higher growth rate [18]. *Hortaea werneckii*, a halophilic yeast (a member of Pezizomycotina), represents another example, wherein a WGD event has contributed towards duplication of a large number of genes to sustain growth under extreme osmotic stress and salt concentrations [40].

It is to be noted that the previous genome-based studies revealed the expansion/emergence of gene families/orthogroups in different AGs of *R. solani* [9, 15]. However, it was not clear whether the gene expansion is due to the potential WGD event or due to mere heterokaryotic, multinucleate nature of the pathogen. Our data suggests that the gene duplication has occurred in AG1-IA after divergence from AG1-IB. We have observed the expansion of gene families in AG1-IA as compared to its closest relative AG1-IB especially, the genes located in the duplicated gene blocks. About 15% of the *R. solani* AG1-IA strain BRS1 genome encompasses the duplicated regions. The duplicated genomic regions are enriched with the pathogenicity-associated gene families, including PHI-base and secreted proteins. We emphasize that WGD, followed by possible sequential duplication events, have contributed towards the evolution of *R. solani* AG1-IA strains. This has empowered *R. solani* AG1-IA to have a vast repertoire of virulence functions that can be deployed to colonize a broad range of plant species. In this regard, it is noteworthy that natural infection by AG1-IA strains of *R. solani* are more profound than the other AGs [41, 42].

We observed that extensive interspersed repeat elements have introduced several breaks in the duplicated gene blocks, due to which the probability of frequent recombination and gene loss as well as modification due to domain loss has increased in *R. solani*. We anticipate that gene duplication events and subsequent gain/loss of functional domain have enabled the *R. solani* to adopt new functions. Indeed, we observed a variation in gene expression pattern of the paralogous gene pairs during infection process, which suggests neofunctionalization of the duplicated genes [43]. Notably, the expression of certain paralogs with the secretion signal or additional domain were significantly lower during pathogenesis in rice, as compared to the corresponding paralogs lacking secretion signal or additional domain. This suggests that *R. solani* may have evolved to downregulate the expression of such paralogs to avoid recognition by the host. Contrastingly, in certain other cases, the paralogs with secretion signal or additional domain showed higher expression during pathogenesis, compared to others that lack secretion signal or additional domain. This emphasizes such paralogs to be involved in promoting successful host colonization. Moreover, our data showed that silencing of one member of the paralogous pairs, but not the other member, compromises the pathogenesis of *R. solani*. This further emphasized that TE-mediated disruption of genes or domain loss has a profound impact on the pathogenesis of AG1-IA.

We analysed the nucleotide diversity in the genome sequence analysis of forty-two field isolates of *R. solani* AG1-IA, collected from different agro-climatic zones of India, and mapped the regions likely to be under purifying and diversifying selection. Interestingly, both the genomic regions with high population mutation rates (*θ⍵*) and with the lowest nucleotide diversity are enriched with TEs. Sequence-based phylogenetic analysis revealed three distinct clades and one admixture of groups I and II. The existence of five isolates as admixtures indicates a natural exchange of genetic materials between the *R. solani* isolates. Although the predominant asexual mode of propagation enables *R. solani* AG1-IA strains to maintain genetic makeup, the ability of strains belonging to same AGs to undergo hyphal fusion (anastomosis) also has been reported [44, 45]. Additionally, a relatively rare sexual mode of propagation (teleomorph: *Thanatephorus cucumeris*) [46, 47] might have contributed towards the exchange of genetic material and development of admixture strains of *R. solani*. It is to be noted that horizontal transfer of lineage-specific genomic regions between the fungal strains can occur at a level of even one-quarter of the whole genome and can lead to acquisition of new functions [48]. The physiological relevance of the exchange of DNA, particularly in terms of the pathogenesis of *R. solani*, needs to be investigated in  future studies.

Several of the genes predicted to be undergoing diversifying selection show induced expression during pathogenesis of *R. solani* in rice emphasizing their importance during infection process. Notably, silencing of one of the highest probable candidates, i.e. *Rs_01468* (encoding GH61, LPMO_AA9 domain-containing protein) significantly compromised the pathogenesis of *R. solani*. The LPMO domain-containing proteins are widespread in filamentous fungi including *R. solani* and enable the pathogens to degrade different components of the host cell wall [9, 49]. In *Colletotrichum*, induction of LPMO has been associated with switching between biotrophic and necrotrophic phases [50]. Considering that *Rs_01468* is induced during 2 and 3 dpi of *R. solani* infection, we anticipate that it may facilitate the transition from biotrophy to necrotrophic phase of pathogenesis in *R. solani*.

We observed 18 genes most likely under purifying selection in *R. solani*, and they encode essential functions such as reverse transcriptase (Rs_06070; Rs_06071), DNA polymerase (Rs_11453), Sir2 family transcriptional regulator (Rs_11455) and glucosamine-phosphate N-acetyltransferase (Rs_11537). The glucosamine 6-phosphate N-acetyltransferase (GNAT) domain-containing proteins play a key role in biosynthesis of chitins in the fungal cell wall [48, 49]. We observed that homologues of GNAT are abundantly present in *R. solani* and that relatively shorter sized homologues (79–87 aa), including *Rs_11537* (79 aa) are predicted to be under purifying selection. Notably, dsRNA-mediated silencing of the *Rs_11537* gene, one of the most probable candidates for being subject to purifying selection, effectively compromised the pathogenesis of *R. solani* in rice as well as tomato. On the other hand, silencing of a relatively larger sized GNAT homologue (Rs_05222; 183 aa) did not compromise the pathogenesis of *R. solani*. This suggests that smaller sized GNAT proteins can be used as novel targets for disease control.

## Conclusions

We propose that a recent whole-genome duplication followed by transposon element-mediated gene loss has shaped the present genomic structure of *R. solani* AG1-IA, an agriculturally important rice pathogen. This has led to an expansion and domain alterations of the gene families associated with its virulence. Genome-wide analysis of multiple field isolates of the pathogen identified genomic regions which are essential for survival and pathogenesis and were, therefore, not allowed to change over the period. The analysis has also identified the regions that are continuously evolving and likely to have been positively selected by nature, enabling the pathogen to adapt to the changing environment and maintain pathogenicity.

## Methods

### Biological materials

*Rhizoctonia solani* AG1-IA strains were grown on Potato Dextrose Agar (PDA; 39 g/L; Himedia, Mumbai, India) plates at 28 °C. The growth rate, maturation of sclerotia, sclerotial size and number were measured for each strain, as described earlier [29]. Also, the pathological attributes of these strains were studied in rice (indica cultivar PB1), and relative vertical sheath colonization (RVSC) was calculated at 3 dpi (days post inoculation), as described earlier [15].

### Genome sequencing and assembly

High-molecular weight DNA was extracted from *R*. *solani* AG1-IA strain BRS1, as described earlier [7], and genomic DNA fragment library was constructed for PacBio SMRT (single molecule real time) sequencing, as per the manufacturer’s instructions. A total of 13.74 Gb (~ 300×) sequence data was generated from two PacBio Sequel runs. Furthermore, two Illumina libraries were prepared with 2 kb and 5 kb insert sizes and a total of 2.99 Gb (2 × 150 base pairs) (~ 67×) sequence data with 2 kb insert size and 3.1 Gb (2 × 150 base pairs) (~ 69×) sequence data with 5 kb insert size were generated.

The de novo assembly of the genome was performed using FALCON and FALCON-Unzip (pb-falcon 0.2.7) [51] tools. FALCON-Unzip tool assembles a set of partially phased primary contigs and fully phased haplotigs. The initial assembly with FALCON was carried out with parameters set, 50 Mb for genome size, 30× for seed coverage and 1000 bp as the length cutoff for seed reads. Furthermore, the FALCON-Unzip module was applied to phase the raw reads according to the SNPs identified in the FALCON assembly and reassemble them in a discrete haplotype-specific manner. The genome assembly was polished and consensus sequences were attained with the Arrow polishing tool in FALCON-Unzip. The Illumina pair-end reads with 5 kb and 2kb insert sizes were used for sequence correction using Pilon v1.23 [52]. First, the reads were mapped to the polished assembly using BWA mem v0.7.17 with -M parameter [53]. Samtools v1.9 [54] was used for indexing, followed by Pilon correction using the parameters “–diploid –fix all” to correct bases, fix misassemblies and fill gaps.

### Genome annotation

The repetitive sequences, identified by RepeatModeller v2.0.1 [55] and Repbase19 database [56], were used to mask *R. solani* genome with RepeatMasker v4.1.0 (http://www.repeatmasker.org/). Three different approaches, i.e. ab initio, homology-based and transcript-based prediction were used to predict the protein-coding genes in the repeat-masked genome assembly. AUGUSTUS v3.3.3 [57] with parameters trained on Coprinus species and GeneMark-ES v4.59 [58] with training data customized for fungus (--max intron 5000) were used for ab initio prediction. For homology-based gene prediction, we used EXONERATE v2.2.0 [59] (with parameters --model p2g --percent 80) and AAT r03052011 [60] (with parameters set for -P --dps ‘-f 100 -i 30 -a 200’ --filter ‘-c 10' --nap ‘-x 10’) tools using the protein sequences predicted from AG1-IA and AG8 draft assemblies. In the transcript-based approach, we aligned AG1-IA transcriptome sequences for spliced alignment using PASA v2.4.1 [61]. The predictions from these three approaches were integrated using EVIDENCEMODELLER (EVM) v1.1.1 [62] to generate consensus gene models. Finally, for the identification of spliced variants and prediction of untranslated regions, the EVM output was run through PASA.

The calculation for the probability of sequential genome duplication was done following the method reported earlier [18]. In case the duplicated regions are created in a sequential manner, those will follow a Poisson distribution in the genome with the formula *f(x;λ)* = *λ*^*x*^*. e*^*-λ*^*/x*!, where *e* = 2.71828, *x* is the probability of which is given by the function and is a positive real number equal to the expected number of occurrences that occur during the given interval. According to this equation, we expect 18.4 triplications per 100 duplicates. Instead of the expected 8 triplications out of 46 duplicates, we have observed 4 triplications. The probability for this observation is *p* (4,8) = 0.057.

### Gene family classification

In order to predict the gene family, proteins derived from genome assemblies of multiple *R. solani* strains belonging to AG1-IA (GCA_016906535.1 [63], GCA_000334115.1 [64], GCA_015342405.1 [65], GCA_015342435.1 [66], GCA_015341985.1 [67], GCA_015342415.1 [68], BRS1), AG1-IB (GCA_000832345.2 [69], GCA_000350255.1 [70]), AG3 (GCA_000715385.1 [71], GCA_000524645.1 [72]), AG8 (GCA_000695385.1 [73]) and AG2-2IIIB (GCA_001286725.1 [74]) were included in the analysis. The full set of proteins for each strain was used to infer gene family (orthogroups) with OrthoFinder v2.4.0 [75]. The programme uses DIAMOND blast with *E*-value < 1e−05 and MCL clustering algorithm with an inflation parameter of 1.5 for the identification of similarity index and clustering. The UpSet diagram was prepared with the R package UpSetR.

### Functional annotation

Sub-cellular localization, secretion status and transmembrane domains were predicted using Phobius v1.01 [76] (https://phobius.sbc.su.se/), SignalP v5.0 (probability ≥ 0.5) [77] (https://services.healthtech.dtu.dk/service.php?SignalP-5.0), TMHMM (selected topology: other) [78] (https://services.healthtech.dtu.dk/service.php?TMHMM-2.0) and TargetP (likelihood of being signal peptide: ≥ 0.5) [79] (https://services.healthtech.dtu.dk/service.php?TargetP-2.0) online tools. The bigPI Fungal Predictor [80] (https://mendel.imp.ac.at/gpi/fungi_server.html) positive score value was used to identify GPI modification sites. The secondary metabolite encoding genes were identified using AntiSMASH fungal version with relaxed detection strictness [81] (https://fungismash.secondarymetabolites.org/#!/start). The putative genes involved in pathogen-host interactions were predicted based on sequence similarity (*E-*value < 10^−5^) in PHI-base (Pathogen Host Interactions Database) [82]. InterProScan v5.28-67.0 [83] was used to assign GO terms and identify conserved domains including fungal-specific transcription factors. The CAZymes encoding genes were predicted using the dbCAN2 meta server (http://bcb.unl.edu/dbCAN2/) by HMMER, DIAMOND and Hotpep-based searches. Effectors were predicted using SignalP v5.0 and EffectorP v2.

### Evolutionary analysis

Syntenic blocks within AG1-IA strains and between AG1-IA and other anastomosis group members were identified using MCScanX [84] with default parameters and synteny distribution was plotted with Circos [85]. Synonymous (*Ks*) and nonsynonymous (*Ka*) substitutions rates of homologous gene pairs were calculated with the perl script, add_ka_and_ks_to_colinearity.pl incorporated in MCScanX. The median *Ks* value for each block was considered to be representative of the duplicated region. *Ks* distribution was plotted to estimate divergence times and genome duplication events. The time was calculated at the peak *Ks* value using the formula *T* = *K*_*s*_/2*r*, where *r* is the fungal neutral substitution rate (*r* = 1.3 × x10^−8^) [24]. The expected number of triplications and the probability of observed triplications is calculated as per the methods described previously [18].

### SNP genotyping and population structure of Indian AG1-IA strains

The high-molecular weight DNA of different *R*. *solani* strains isolated from different parts of India (Table S15) was extracted, as described earlier [7] and subjected to Illumina (Novaseq) sequencing (2 × 150 bp), as per the manufacturer’s instructions. Around 16 Gb (~ 350×) sequence data was generated for each of the strains and low-quality reads (PHRED score < 20 and length < 30 bp) were trimmed initially. The trimmed reads were mapped to AG1-IA reference genome with BWA v0.7.17-r1188 mem with -M and analysed for PCR duplicates with MarkDuplicates in Picard v2.23.3 (https://broadinstitute.github.io/picard/). The SAM files were sorted, indexed and converted to BAM format with Samtools v1.9. Using the Genome Analysis Toolkit (GATK v4.1.8.1 [86], variants were initially identified by HaplotypeCaller with option -ERC GVCF, and then combined genotyping was performed with GenotypeGVCFs. SNP variants were selected with SelectVariants and filtered for QD < 2.0, FS > 60.0, MQ < 40.0, MQRankSum < − 12.5, ReadPosRankSum < − 8.0 and SOR > 4.0. The variants were annotated by SnpEff [87] based on the annotation GFF file. Principal component analysis (PCA) was conducted with TASSEL v5 [88], and the result was plotted with ggplot2. A phylogenetic neighbour-joining tree was generated from the numerical genotype data using TASSEL and visualized with iTOL (interactive tree of life https://itol.embl.de/). The average pairwise divergence among genotypes or observed nucleotide diversity (*π*), expected nucleotide diversity or estimated mutation rate (*θ*) and Tajima’s *D* was calculated with TASSEL using default settings. The genomic regions representing the 1st quantile of the upper and lowermost Tajima’s *D* statistic were considered as the candidate regions under selection. The population structure was determined by ADMIXTURE v1.3.0 [89] with *K* varying from 2 to 5.

### Development of *R. solani* genome database

A rice sheath blight (RSB) database has been developed to host the genome assembly and annotation of *R*. *solani* AG1-IA genome. The database operates on a linux system and can be assessed by the URL: https://nipgr.ac.in/RSB/. The current database framework is built on the Apache server, and the RSB web interface has been designed with laravel (https://laravel.com/), an open-source PHP framework, HTML (https://html.spec.whatwg.org/), CSS (https://www.w3.org/Style/CSS/Overview.en.html) and JavaScript (https://www.javascript.com/). In addition, RSB is integrated with stand-alone BLAST [90] for online similarity search against genome, proteome, CDS or mRNA of any anastomosis groups of *R*. *solani*; jBrowse [91] for interactive visualization of the genome; and batch download for genome, mRNA, CDS and protein.

### Phylogenetic analysis

Annotated GNAT proteins in *R. solani* were identified and extracted from NCBI and used to find members in AG1-IA assembly based on homology. The sequences were examined for the GNAT family domains and classified accordingly. Multiple sequence alignment of the protein sequences was performed using CLUSTALW. The best model for the phylogenetic tree reconstruction was predicted with ProTest v3.4.2, and the maximum likelihood tree with 1000 bootstraps was constructed with MEGAX using the JTT+G_F model [92].

### Pathological assays

For infection in rice (indica cultivar PB1), freshly grown equal sized *R. solani* strain BRS1 sclerotia were inoculated within the sheaths of ~ 45 days old rice plants. The disease symptoms were quantified in terms of relative vertical sheath colonization (RVSC) as described earlier [15]. The infected tissues (including 1 cm up and down from the site of infection) were harvested for expression analysis at 0, 1, 2, 3 and 4 dpi. A total of 3–4 sheaths per plant were infected, and a total of 4–5 plants were used per experiment.

In tomatoes, the *R. solani* sclerotia were attached to the abaxial surface of the leaves of 30-day-old tomato plants (*Solanum lycopersicum*; Pusa Ruby) using aluminium strips [93]. The plants were further incubated in a growth chamber at 26 °C temperature under 80% relative humidity and 12/12 h of day/night cycle. The disease symptoms were recorded at 3 dpi, and disease severity was categorized into severe, medium and negligible symptoms. A total of 4–5 plants and three leaves per plants were infected with *R. solani*, and each experiment was repeated three times.

### qRT-PCR-based expression analysis and pathogen load quantification

The qRT-PCR-based expression analysis of *R. solani* genes was carried out during pathogenesis in rice (cv. PB1) and tomato (cv. Pusa Ruby). The primers were designed to selectively amplify the target pathogen genes (Additional file 19: Table S18), and qRT-PCR was performed using Sso Advanced Universal SYBR Green Supermix (BioRad) according to the manufacturer’s instructions. The relative expression was calculated using the 2^−ΔCt^ method, wherein ΔCt is the difference between Ct values of target and reference (*18S rRNA*) genes of *R. solani* [94]. The fold change was calculated using the 2^−ΔΔCt^ method [95] wherein ^ΔΔ^Ct is the difference in the expression of *R. solani* genes at 1 dpi, 2 dpi, 3 dpi and 4 dpi, compared to 0 dpi. The fungal load was quantified in the infected samples, using the 2^−ΔCt^ method, wherein ΔCt is the difference between Ct values of fungal *18S rRNA* (target) and host *actin* gene (as reference), as described earlier [93]. The data from three independent biological replicates were used to calculate the standard error, and one-way ANOVA was performed using the Sigma Plot 11.0 (SPSS, Inc. Chicago, IL, USA) software to test the statistical significance (determined at *p* ≤ 0.05) between separate groups using the Student-Newman-Keuls test.

### dsRNA mediated silencing of *R. solani* genes and functional studies

For dsRNA mediated silencing of *R. solani* genes, the sequences were analysed using the siFi21 software (http://labtools.ipk-gatersleben.de/) [96] to select unique fragments with no off-target silencing effect in *R. solani* as well as host (rice/tomato) genome. The target regions were PCR amplified from the *R. solani* cDNA using gene-specific primer pairs having T7 promoter sequence at the 5′ end of the forward primer (Additional file 19: Table S18). For in vitro transcription, 1 μg of the purified gene fragments was used to produce dsRNA, as per the manufacturer’s protocol (using MEGAscript T7 Transcription Kit; Thermo Scientific). The amount of dsRNA synthesized was measured using Nanodrop (Themo Scientific), and 50 μg of dsRNA was used to treat *R. solani* sclerotia. The dsRNA-treated *R. solani* were inoculated in the tillers of rice cv. PB1 (45 days old) or in the leaves of tomato cv. Pusa Ruby plants (~ 30 days old), as described before [15, 29]. The plants were incubated in the infection chamber at 28 °C, and disease symptoms were recorded at 3 dpi. The gene silencing was investigated using qRT-PCR, following the protocol described earlier [29]. The list of primers used in the study is enlisted in Additional file 19: Table S18. For rice, at least 5 plants (4 tillers each) were analysed for each treatment, and experiments were repeated three times. Relative vertical sheath colonization based disease index was estimated and pathogen load was calculated, as described previously [93]. For tomato, at least five plants (three leaves per plant) were analysed for each treatment, and the experiment was independently repeated three times (biological replicates). The disease index and pathogen load were estimated, as described before [93].

## Supplementary Information

**Additional file 1: Table S1.** Summary statistics of *R. solani* AG1-IA strain BRS1 genome assembly.

**Additional file 2: Table S2.** Statistics of repeat elements in the *R. solani* AG1-IA strain BRS1 genome.

**Additional file 3: Table S3.** Annotation statistics of *R. solani* AG1-IA strain BRS1 genome.

**Additional file 4: Table S4.** Predicted pathogenicity-associated genes in *R. solani* AG1-IA strain BRS1 and GO-classification.

**Additional file 5: Table S5:** Secondary metabolite encoding gene clusters in *R. solani* AG1-IA strain BRS1.

**Additional file 6: Fig. S1.** Rice sheath blight (RSB) database. A. Blast search interface; B. Jbrowser for genome visualization. **Fig. S2.** Synteny between R. solani AG1-IA strain BRS1 and A. *R. solani* AG1-IA strain XN, B. *R. solani* AG1-IA strain B2, genomes. **Fig. S3.** Synteny between AG1-IA and A. AG8; B. AG3; C. AG2-2IIIB; D. AG1-IB genome assemblies. **Fig. S4.** UpSet diagram showing distribution of gene orthogroups among the genome assemblies of A. *R*. *solani* AG1-IA strains (BRS1, XN and B2) and B. different *R*. *solani* anastomosis groups. **Fig. S5.** Expression profile of selected *R. solani* AG1-IA strain BRS1 unique genes during pathogenesis in rice (PB1). **Fig. S6.** Depiction of four triplicated paralogous blocks of AG1-IA genome. **Fig. S7.** Circos images showing the duplicated syntenic blocks in the genome assemblies of A. *R. solani* AG1-IA strain XN and B. *R. solani* AG1-IA strain B2. C. Plot of Ks vs. paralogous gene pairs of *R. solani* AG1-IA strain XN and *R. solani* AG1-IA strain B2. **Fig. S8.** Expression profile of selected *R. solani* AG1-IA paralogous gene pairs during pathogenesis in rice (PB1). **Fig. S9.** qRT-PCR based expression analysis reflecting effective silencing of target genes upon infection with gene specific dsRNA treated *R. solani* in A, C rice and B, D tomato, at 3 dpi. **Fig. S10.** Distribution of *R. solani* isolates as per different agro-climatic zones of India. **Fig. S11.** Classification of the Indian rice field isolates of *R. solani* AG1-IA based on their genomic diversity. A. Unrooted dendrogram depicting the genetic relationship among the isolates. B. Principle component analysis of different isolates clustered them into 3 major groups and an admixture group. **Fig. S12.** Expression analysis of *R. solani* AG1-IA genes under diversifying selection during pathogenesis in rice (PB1). **Fig. S13.** Phylogenetic analysis of *R. solani* GNAT gene family. **Fig. S14.** qRT-PCR based expression analysis of *R. solani* genes during pathogenesis at 3 dpi. A and B reflect the effective silencing of target genes upon infection with gene-specific dsRNA-treated *R. solani* in rice and tomato, respectively*.*

**Additional file 7: Table S6.** Orthogroups and gene count in *R. solani* AG1-IA strains.

**Additional file 8: Table S7.** Orthogroups and gene count in different AGs of *R. solani*.

**Additional file 9: Table S8.** RNA-Seq based expression analysis of *R. solani* strain BRS1 genes.

**Additional file 10: Table S9.** List of genes present in 46 pairs of duplicated syntenic gene blocks in *R. solani* strain BRS1.

**Additional file 11: Table S10.** Mapping statistics of PacBio SMRT long read, Illumina short reads and RNA-Seq reads on the BRS1 contigs hosting duplicated syntenic blocks.

**Additional file 12: Table S11.** Orthologs of AG1-IA strain BRS1 paralogous gene pairs in the AG1-IB genome assembly.

**Additional file 13: Table S12.** TE-associated paralogs of BRS1 having Ka/Ks> 1.

**Additional file 14: Table S13.** Secreted and non-secreted effector paralogs in BRS1.

**Additional file 15: Table S14.** Variation in domain architecture of paralogous genes in BRS1.

**Additional file 16: Table S15.** Passport data and pathogenicity of *R. solani* AG1-IA isolates collected from rice fields across India.

**Additional file 17: Table S16.** Grouping of *R. solani* AG1-IA strains based upon population genetic structure.

**Additional file 18: Table S17.** Genes under diversifying and purifying selection in *R. solani* AG1-IA Indian isolates.

**Additional file 19: Table S18.** List of primers used in the study.

## Data Availability

All data presented in this study are included in the article, its supplementary information files (tables and figures) and publicly available repositories. The present *R. solani* AG1-IA genome assembly and protein-coding gene annotation are submitted to the database of the National Center for Biotechnology Information (NCBI) with a bioproject ID PRJNA715598 [97] and hosted in a web-based user-interactive rice sheath blight (RSB) database (http:nipgr.ac.in/RSB). In addition, all data including vcf files are publicly accessible via Figshare [98].

## Abbreviations

TE: Transposable elements
AG: Anastomosis group
BLAST: Basic Local Alignment Search Tool
dsRNA: Double-stranded RNA
WGD: Whole-genome duplication
GNAT: Glucosamine 6-phosphate N-acetyltransferase
LPMO_AA9: Lytic polysaccharide monooxygenase_Auxillary activity family 9 domain containing proteins
