# Supplementary material for: Evolution of pathogenicity-associated genes in Rhizoctonia solani AG1-IA by genome duplication and transposon-mediated gene function alterations
Source: BMC Biol. 2023 Feb 1;21:15. doi: 10.1186/s12915-023-01526-0 (PMC9890813; doi:10.1186/s12915-023-01526-0)
Supplement: Supplementary file 6 — Additional file 6: Fig. S1. Rice sheath blight (RSB) database. A. Blast search interface; B. Jbrowser for genome visualization. Fig. S2. Synteny between R. solani AG1-IA strain BRS1 and A. R. solani AG1-IA strain XN, B. R. solani AG1-IA strain B2, genomes. Fig. S3. Synteny between AG1-IA and A. AG8; B. AG3; C. AG2-2IIIB; D. AG1-IB genome assemblies. Fig. S4. UpSet diagram showing distribution of gene orthogroups among the genome assemblies of A. R. solani AG1-IA strains (BRS1, XN and B2) and B. different R. solani anastomosis groups. Fig. S5. Expression profile of selected R. solani AG1-IA strain BRS1 unique genes during pathogenesis in rice (PB1). Fig. S6. Depiction of four triplicated paralogous blocks of AG1-IA genome. Fig. S7. Circos images showing the duplicated syntenic blocks in the genome assemblies of A. R. solani AG1-IA strain XN and B. R. solani AG1-IA strain B2. C. Plot of Ks vs. paralogous gene pairs of R. solani AG1-IA strain XN and R. solani AG1-IA strain B2. Fig. S8. Expression profile of selected R. solani AG1-IA paralogous gene pairs during pathogenesis in rice (PB1). Fig. S9. qRT-PCR based expression analysis reflecting effective silencing of target genes upon infection with gene specific dsRNA treated R. solani in A, C rice and B, D tomato, at 3 dpi. Fig. S10. Distribution of R. solani isolates as per different agro-climatic zones of India. Fig. S11. Classification of the Indian rice field isolates of R. solani AG1-IA based on their genomic diversity. A. Unrooted dendrogram depicting the genetic relationship among the isolates. B. Principle component analysis of different isolates clustered them into 3 major groups and an admixture group. Fig. S12. Expression analysis of R. solani AG1-IA genes under diversifying selection during pathogenesis in rice (PB1). Fig. S13. Phylogenetic analysis of R. solani GNAT gene family. Fig. S14. qRT-PCR based expression analysis of R. solani genes during pathogenesis at 3 dpi. A and B reflect the effective silenci [file 12915_2023_1526_MOESM6_ESM.pdf]

A

B

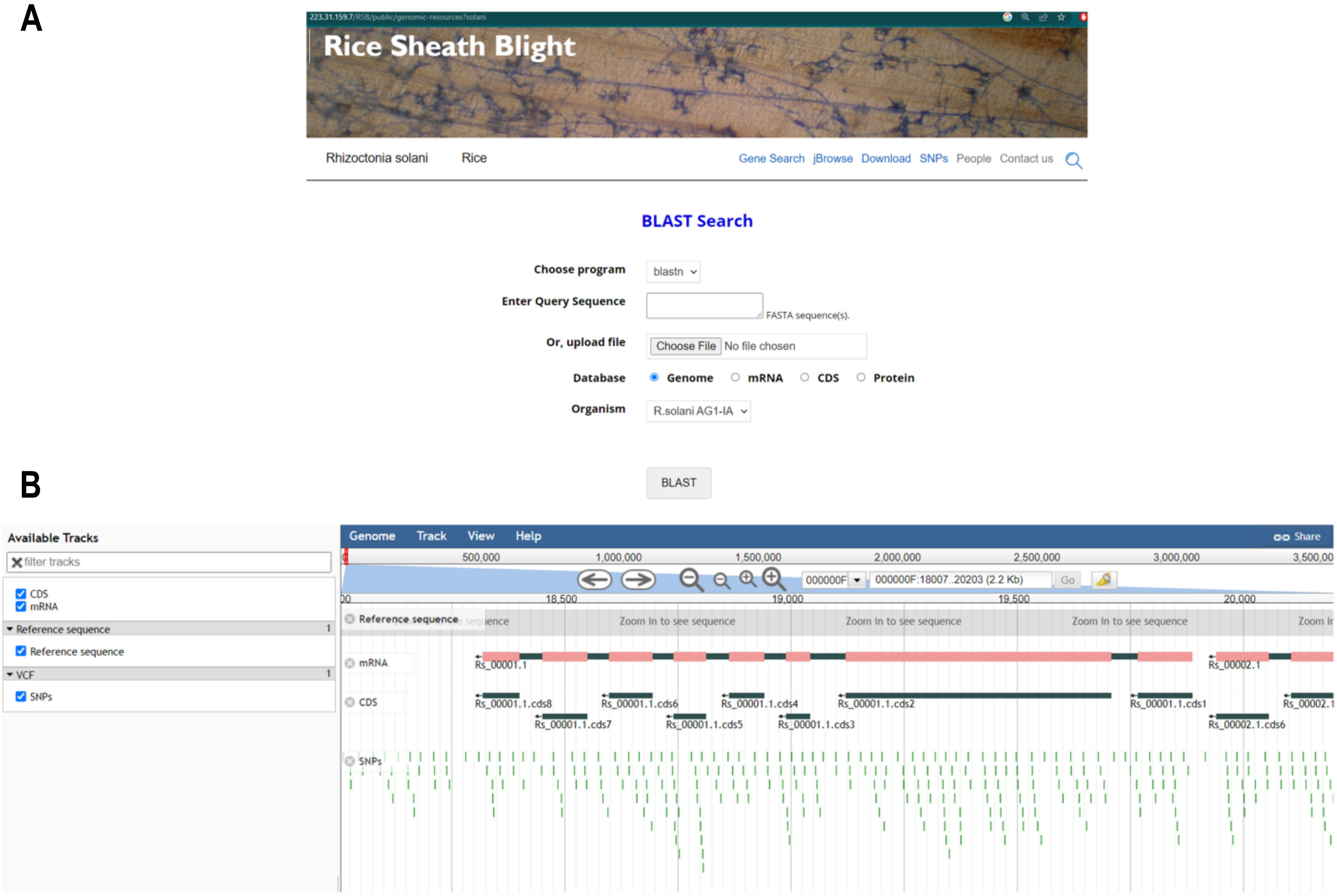

Fig. S1. Rice sheath blight (RSB) database. **A.** Blast search interface; **B**. Jbrowser for genome visualization

A.

B.

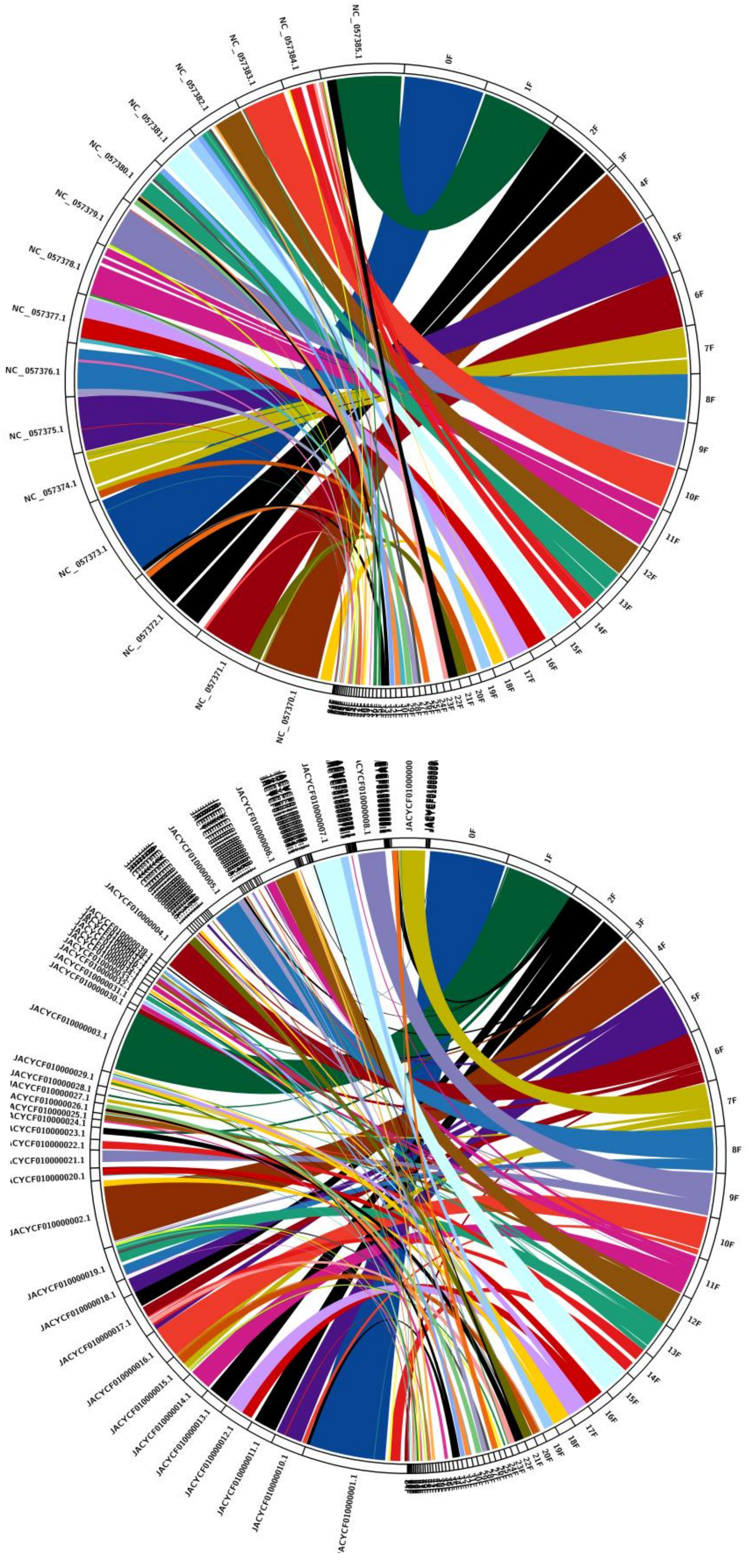

Fig. S2. Synteny between *R. solani* AG1-IA strain BRS1 and **A**. *R. solani* AG1-IA strain XN, **B.** *R. solani* AG1-IA strain B2, genomes.

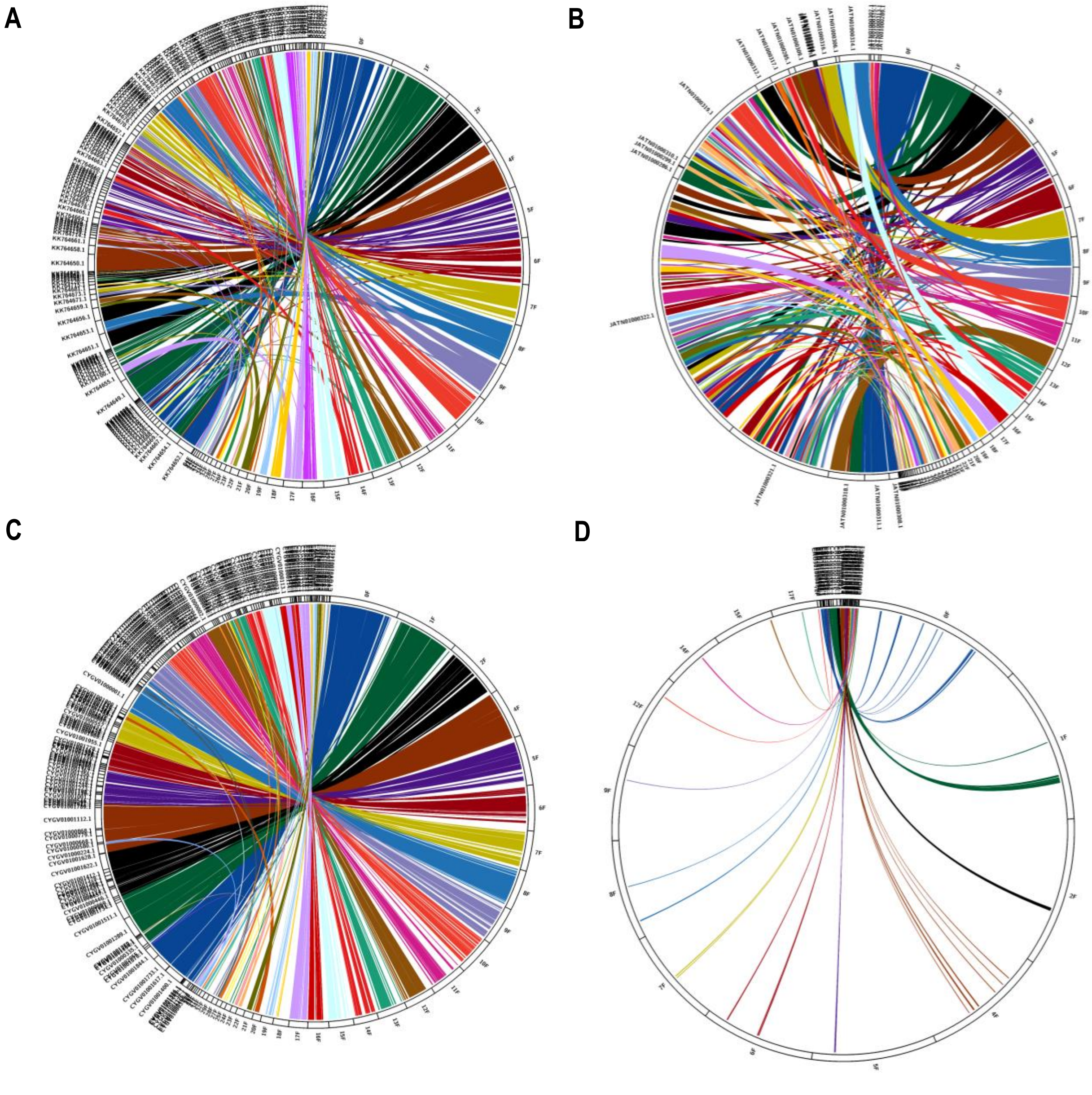

Fig. S3. Synteny between AG1-IA and **A**. AG8; **B.** AG3; **C**. AG2-2IIIB; **D.** AG1-IB genome assemblies.

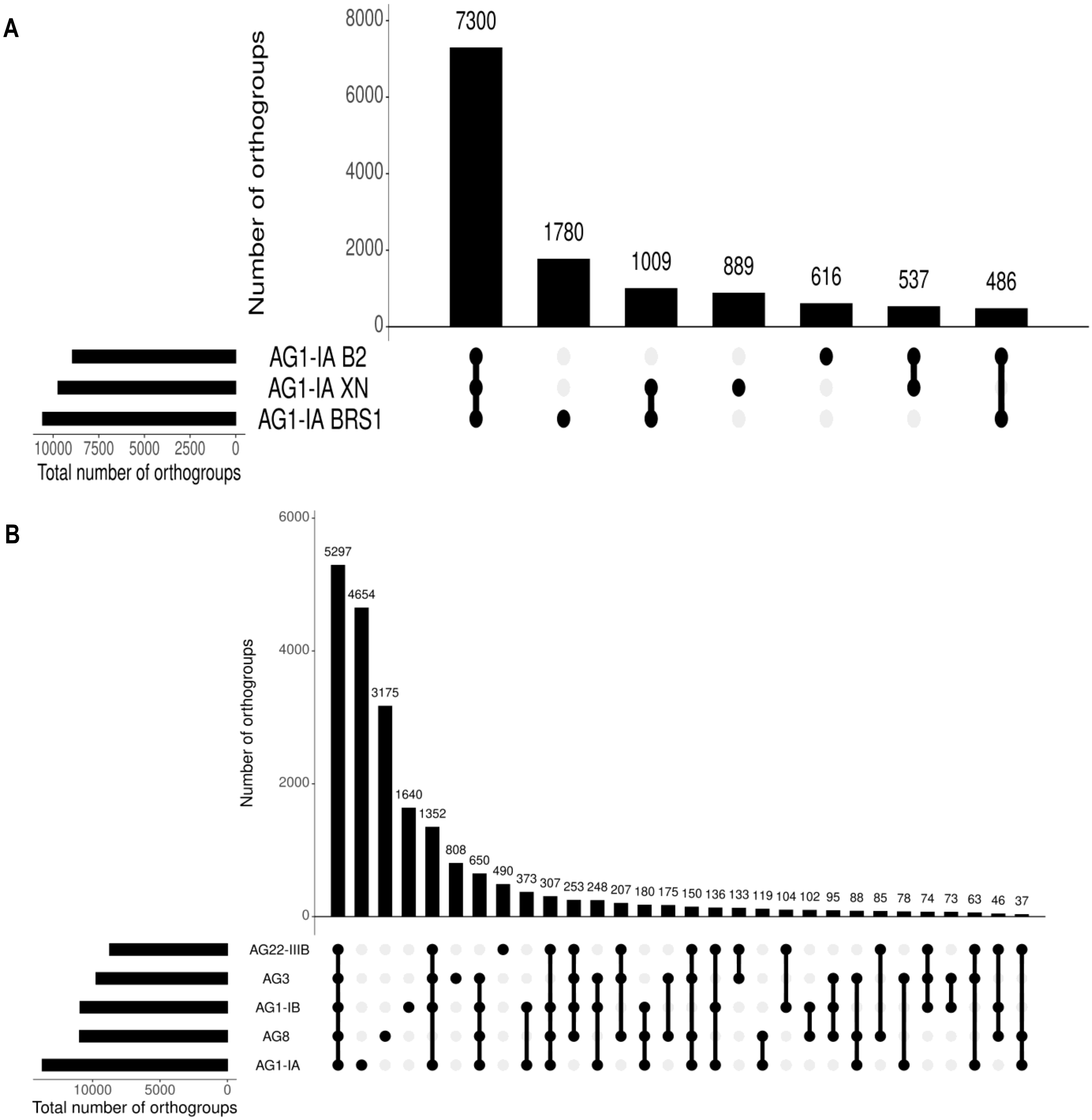

Fig. S4. UpSet diagram showing distribution of gene orthogroups among the genome assemblies of **A.** *R. solani* AG1-IA strains (BRS1, XN and B2) and **B.** different *R. solani* anastomosis groups.

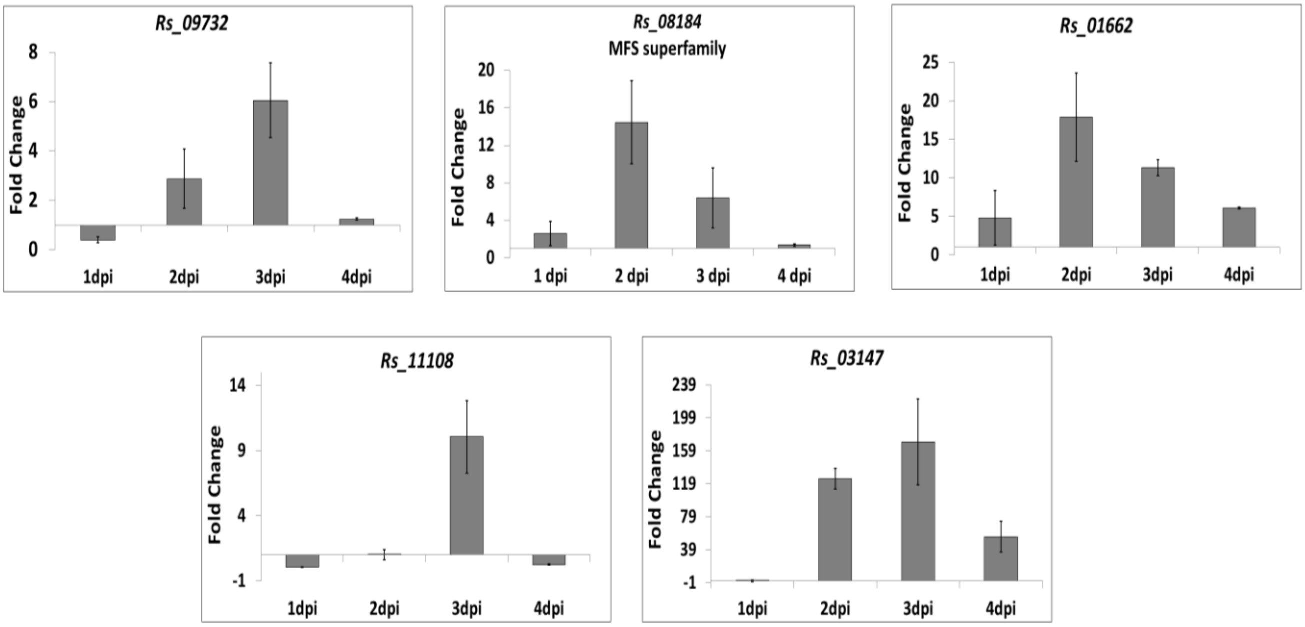

Fig. S5. Expression profile of selected *R. solani* AG1-IA strain BRS1 unique genes during pathogenesis in rice (PB1). The gene expression at indicated time points was quantified with respect to 0 dpi samples, using 18S rRNA as an endogenous control. Data represents mean value of three biological replicates and error bars indicates standard error of the mean. MFS= Major Facilitator Superfamily.

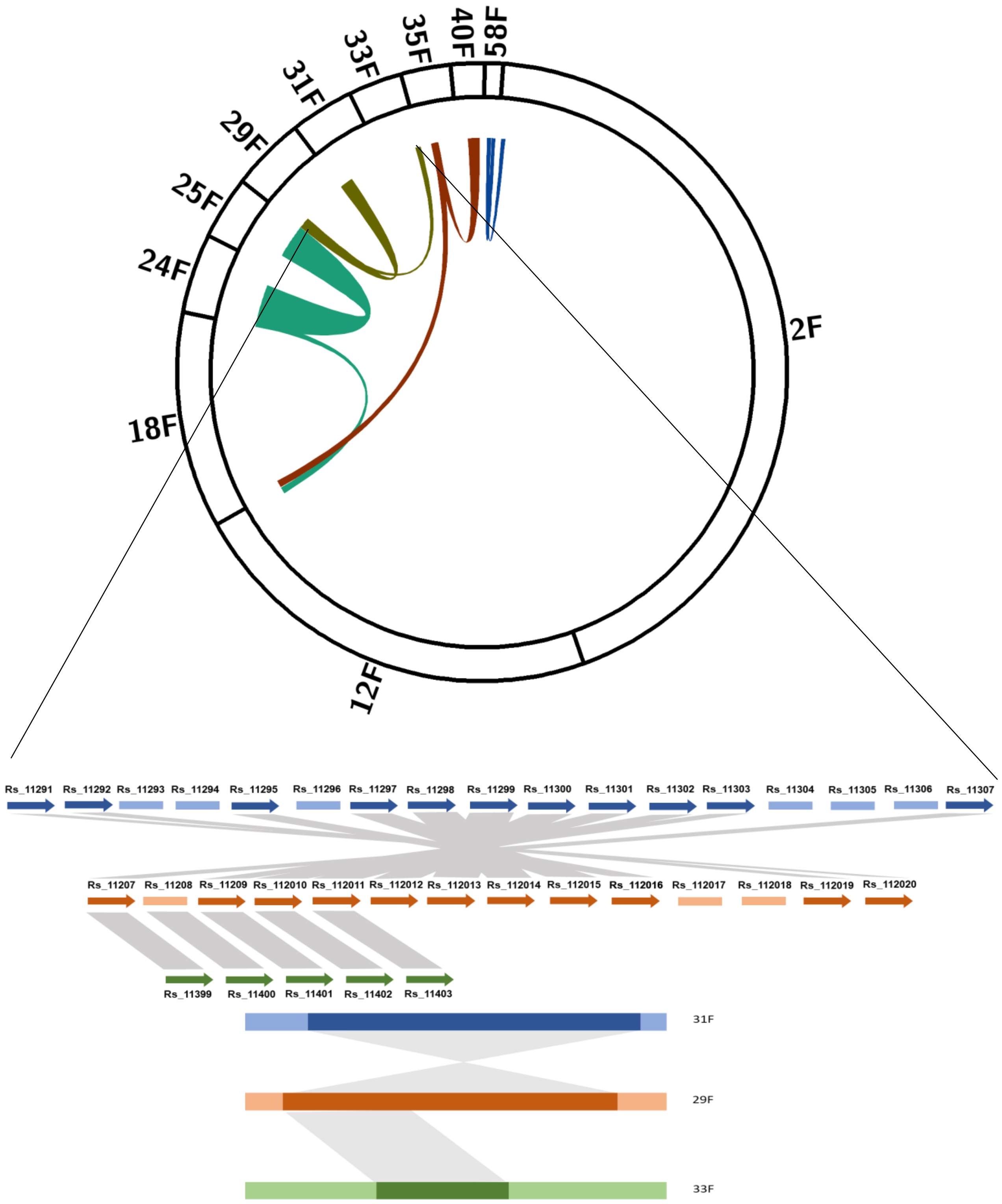

Fig. S6. Depiction of four triplicated paralogous blocks of AG1-IA genome.

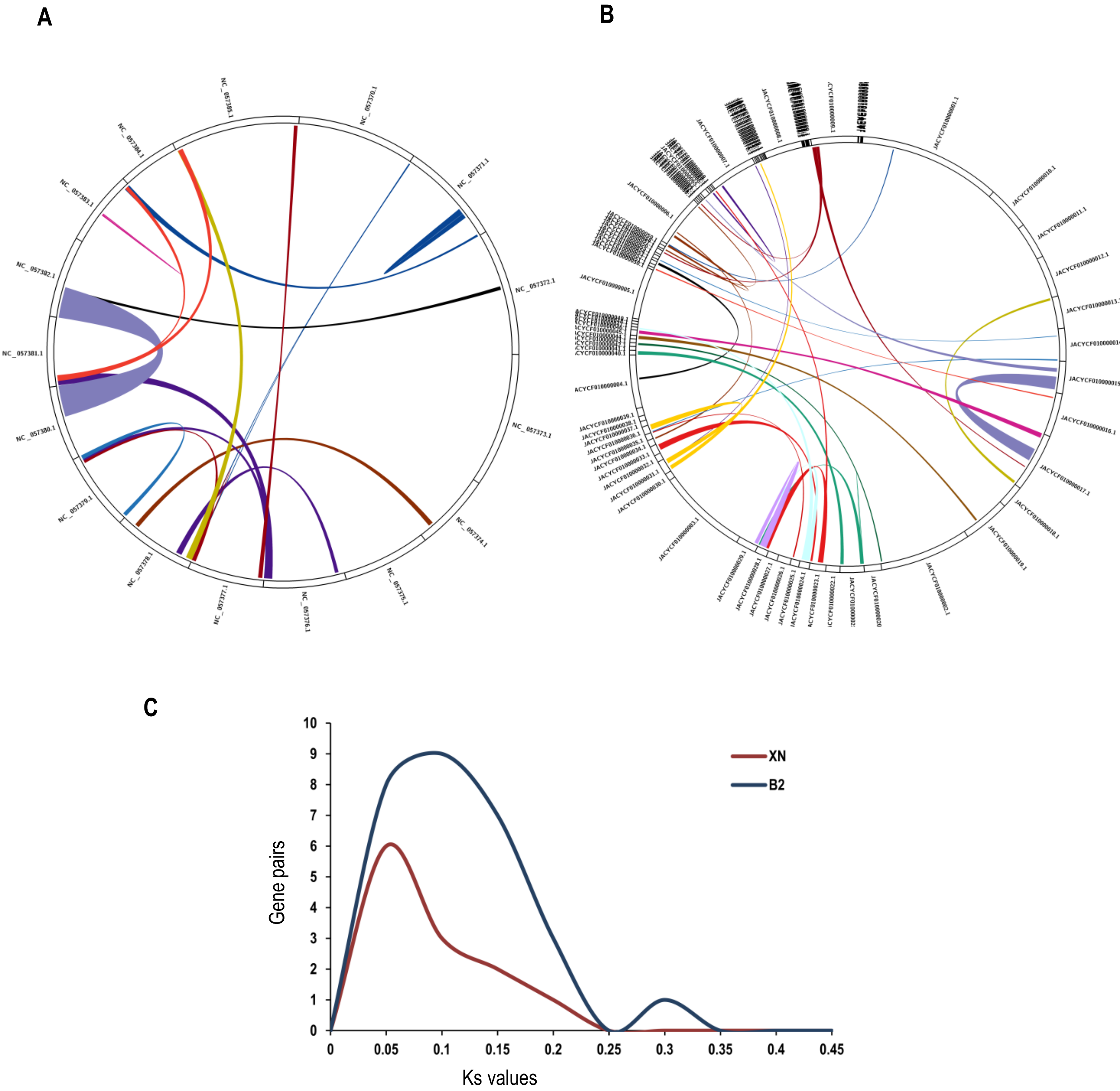

Fig. S7. Circos images showing the duplicated syntenic blocks in the genome assemblies of **A.** *R. solani* AG1-IA strain XN and **B.** *R. solani* AG1-IA strain B2. **C**. Plot of Ks vs. paralogous gene pairs of *R. solani* AG1-IA strain XN and *R. solani* AG1-IA strain B2.

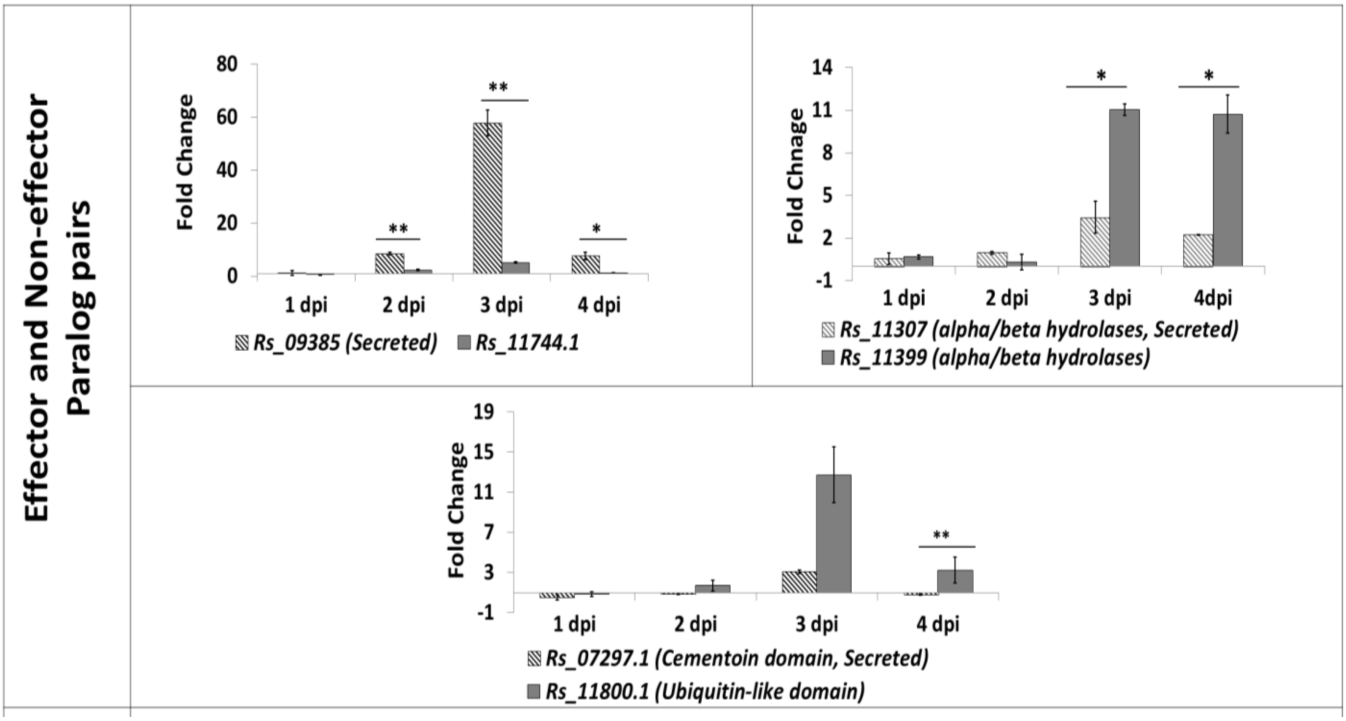

Fig. S8. Expression profile of selected *R. solani* AG1-IA paralogous gene pairs during pathogenesis in rice (PB1). The paralogous pairs were categorized as effector or non-effector (presence or absence of predicted secretory signal). The expression at indicated time points was quantified with respect to 0 dpi samples using 18S rRNA of *R. solani* as endogenous control. Data represent the mean value of three biological replicates and error bars indicate the standard error of the mean.

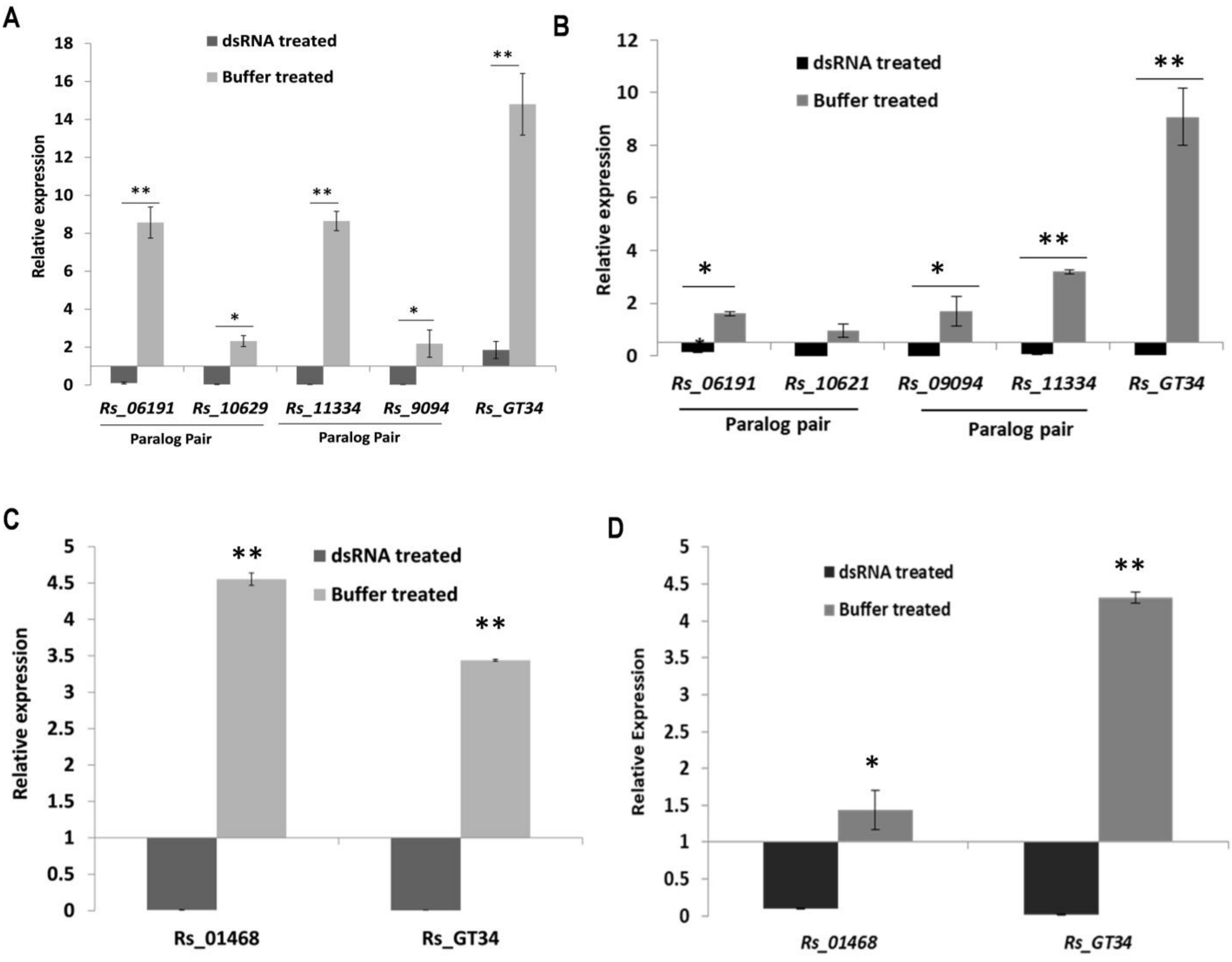

Fig. S9. qRT-PCR based expression analysis reflecting effective silencing of target genes upon infection with gene specific dsRNA treated *R. solani* in **A, C** rice and **B, D** tomato, at 3 dpi. The relative expression of the target genes was estimated using 18S rRNA of *R. solani*, as endogenous control. Data represents mean values ± standard error of three biological replicates.

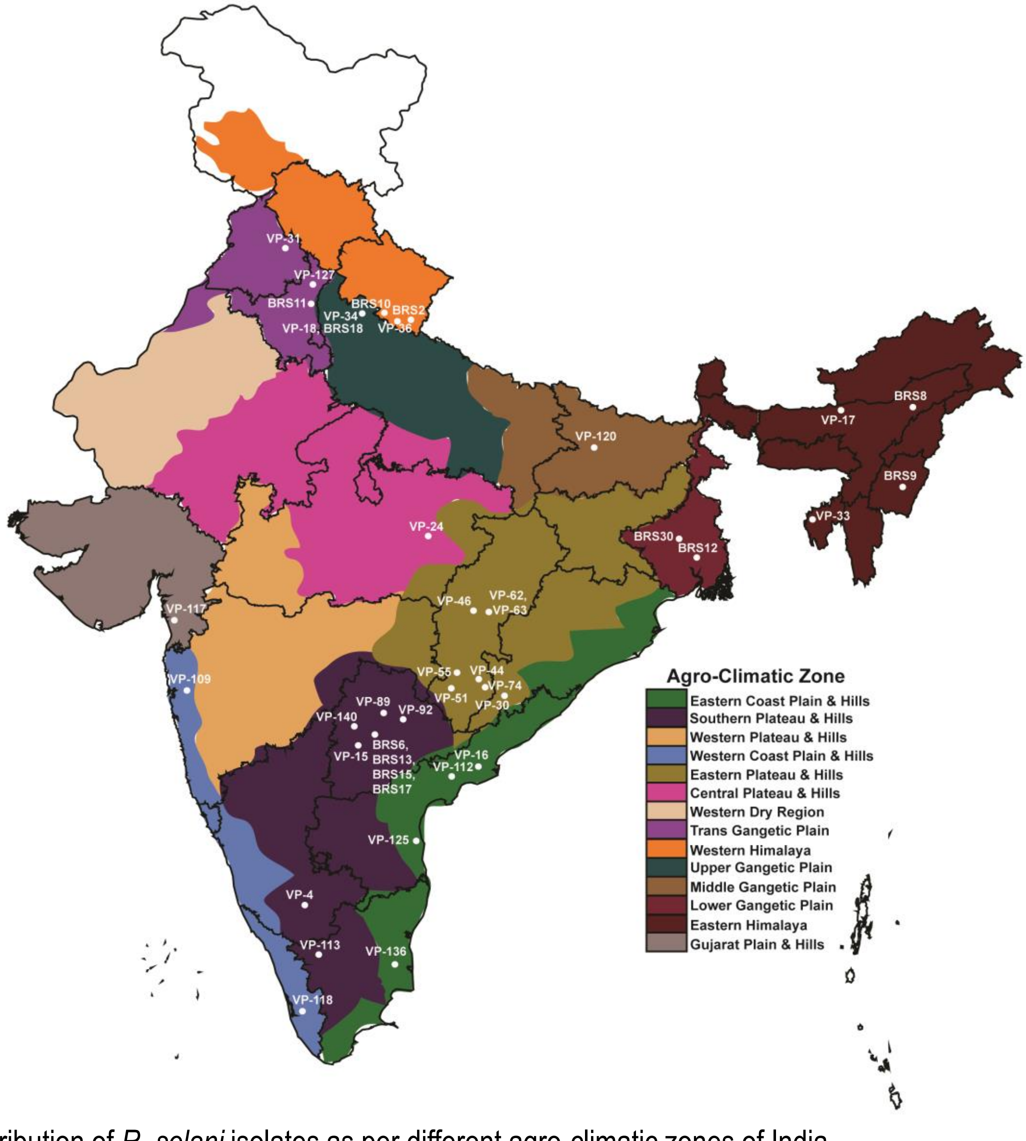

Fig. S10. Distribution of *R. solani* isolates as per different agro-climatic zones of India.

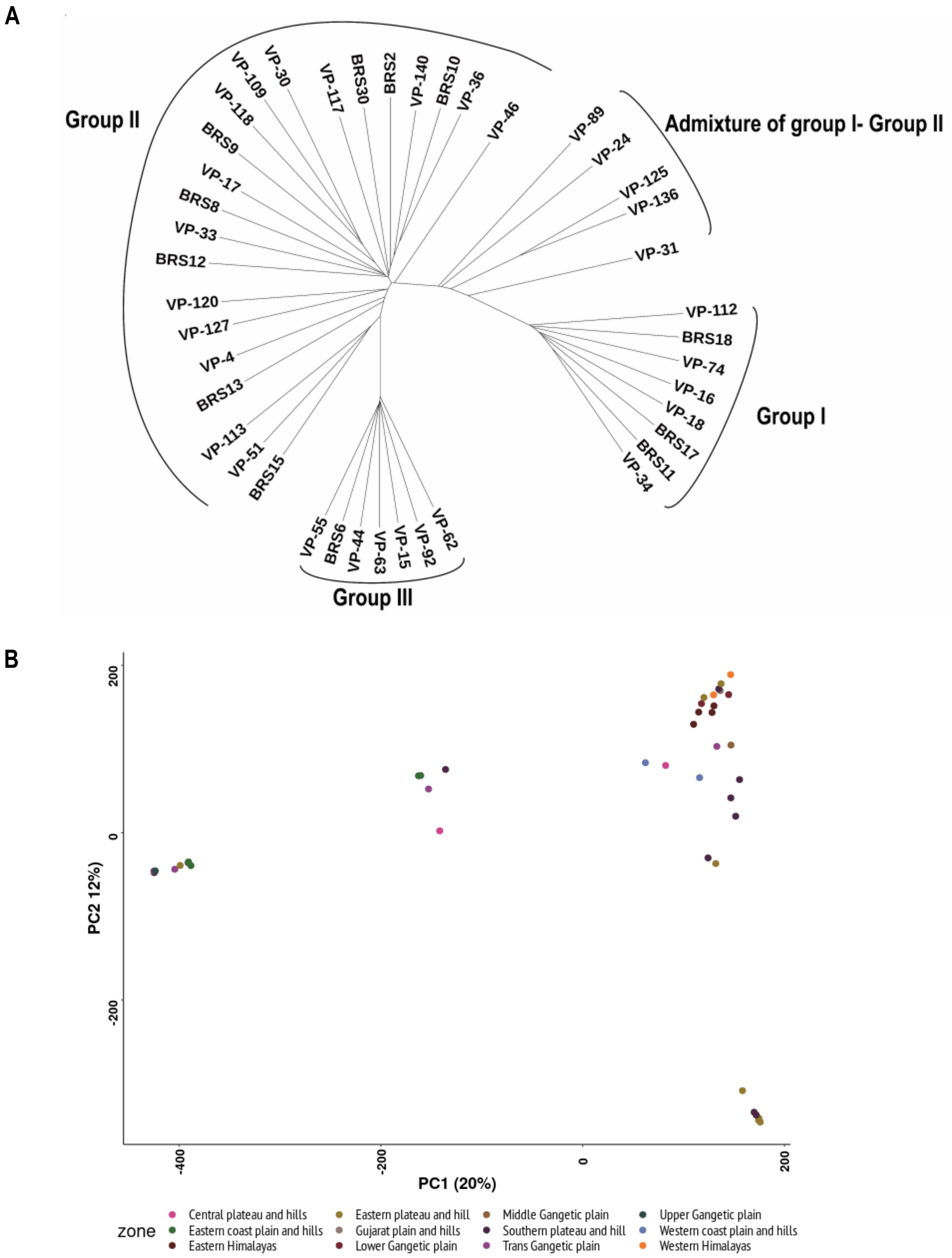

Fig. S11. Classification of the Indian rice field isolates of *R. solani* AG1-IA based on their genomic diversity. **A**. Unrooted dendrogram depicting the genetic relationship among the isolates. **B.** Principle component analysis of different isolates clustered them into 3 major groups and an admixture group.

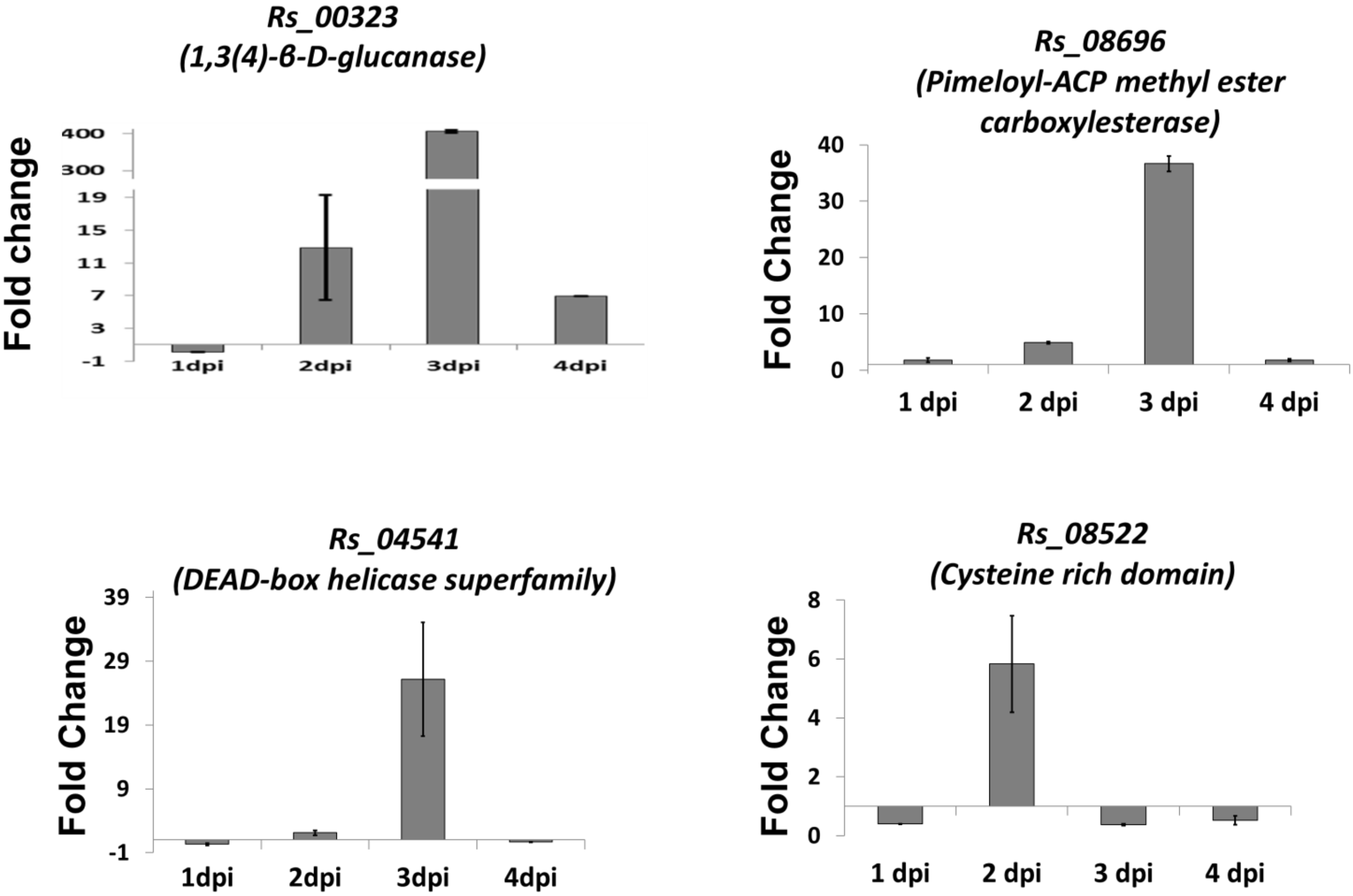

Fig. S12. Expression analysis of *R. solani* AG1-IA genes under diversifying selection during pathogenesis in rice (PB1). The expression at indicated time points was quantified with respect to 0 dpi samples using 18S rRNA as endogenous control. The predicted molecular function/ domain is indicated for each gene. The genes were categorized under three different categories based on their predicted molecular function. Data represent the mean value of three biological replicates and error bars indicate the standard error of the mean. LPMO=Lytic polysaccaharide monoxygenase, ACP=Acyl carrier protein.

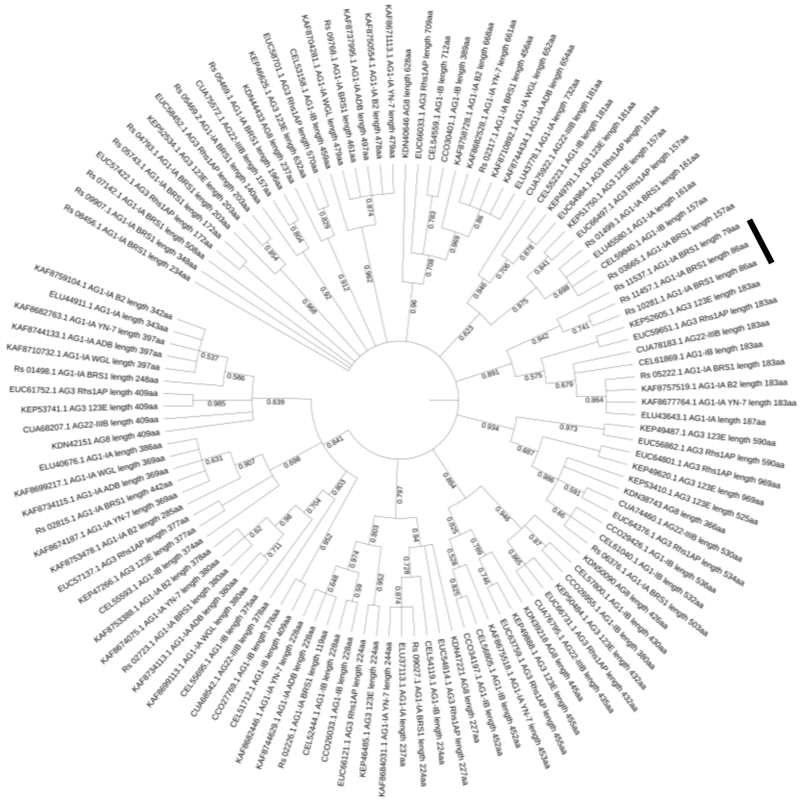

Fig. S13. Phylogenetic analysis of *R. solani* GNAT gene family. Unrooted phylogenetic tree of GNAT proteins constructed by neighbor joining method with 1000 bootstraps using MEGA X tool. Numbers on branches represent bootstrap values (based on 1000 replications), only the values ≥50% are presented. The clade having smaller sized GNAT proteins in *R. solani* AG1-IA strain BRS1 is marked.

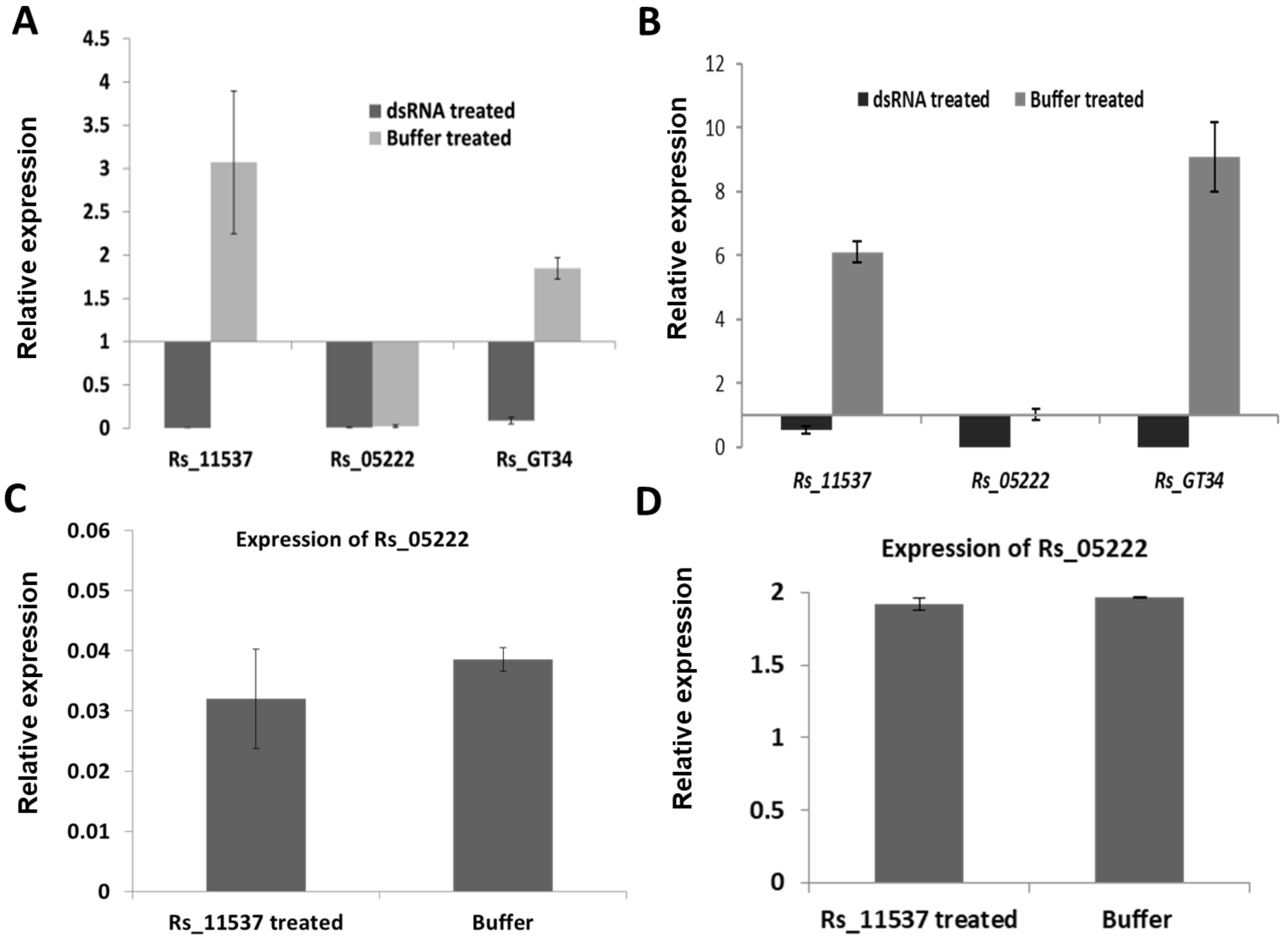

Fig. S14. qRT-PCR based expression analysis of *R. solani* genes during pathogenesis at 3 dpi. **A** and **B** reflect the effective silencing of target genes upon infection with gene-specific dsRNA-treated *R. solani* in rice and tomato, respectively. The expression of the non-target gene (*Rs_05222*) remains unaltered during the pathogenesis of *Rs_11537*-silenced *R. solani,* in **C** rice and **D** tomato. The relative gene expression was estimated using 18S rRNA of *R. solani*, as endogenous control. Data represent mean values ± standard error of three biological replicates.
